# Exploring the Therapeutic Potential of *Ammodaucus leucotrichus* Seed Extracts: A Multi-Faceted Analysis of Phytochemical Composition, Anti-Inflammatory Efficacy, Predictive Anti-Arthritic Properties, and Molecular Docking Insights

**DOI:** 10.3390/ph17030385

**Published:** 2024-03-18

**Authors:** Cheima Djehiche, Nadia Benzidane, Hanene Djeghim, Mehdi Tebboub, El Hassen Mokrani, Saad Mebrek, Mohammed Messaoudi, Chawki Bensouici, Ali Alsalme, David Cornu, Mikhael Bechelany, Lekhmici Arrar, Ahmed Barhoum

**Affiliations:** 1 Laboratory of Applied Biochemistry, Department of Biochemistry, Faculty of Nature and Life Sciences, Ferhat Abbas University of Setif 1, Setif 19000, Algeria; djehichecheima@gmail.com (C.D.); nadia19000@hotmail.fr (N.B.); 2 Biochemistry Laboratory, Division of Biotechnology and Health, Biotechnology Research Center (CRBt), Constantine 25000, Algeria; livenanou@hotmail.com (H.D.); saad.mebrek@gmail.com (S.M.); chawkiislam@yahoo.fr (C.B.); 3 Department of Mechanical Engineering, Faculty of Science and Technology, University Mentouri Brothers Constantine 1, Constantine 25000, Algeria; mehdi.tebboub9@gmail.com; 4 Laboratory of Applied Biochemistry, Department of Biochemistry and Cellular and Molecular Biology, Faculty of Natural and Life Sciences, University Mentouri Brothers Constantine 1, Constantine 25000, Algeria; mohsenmokrani@hotmail.fr; 5 Nuclear Research Centre of Birine, P.O. Box 180, Ain Oussera 17200, Algeria; messaoudi2006@yahoo.fr; 6 Department of Chemistry, College of Science, King Saud University, Riyadh 11451, Saudi Arabia; aalsalme@ksu.edu.sa; 7 Institut Européen des Membranes (IEM), UMR 5635, Univ. Montpellier, ENSCM, CNRS, Place Eugène Bataillon, 34095 Montpellier, France; david.cornu@umontpellier.fr (D.C.); mikhael.bechelany@umontpellier.fr (M.B.); 8 Functional Materials Group, Gulf University for Science and Technology (GUST), Mubarak Al-Abdullah 32093, Kuwait; 9 NanoStruc Research Group, Chemistry Department, Faculty of Science, Helwan University, Cairo 11795, Egypt

**Keywords:** protein denaturation inhibition, anti-proteinase activity, rheumatoid arthritis, GC–MS analysis, phytoconstituents, in silico docking, molecular docking analysis, trypsin inhibition, 2-hydroxyacetohydrazide

## Abstract

*Ammodaucus leucotrichus* exhibits promising pharmacological activity, hinting at anti-inflammatory and anti-arthritic effects. This study investigated seed extracts from *Ammodaucus leucotrichus* using methanol and n-hexane, focusing on anti-inflammatory and anti-arthritic properties. The methanol extract outperformed the n-hexane extract and diclofenac, a reference anti-inflammatory drug, in trypsin inhibition (85% vs. 30% and 64.67% at 125 μg/mL). For trypsin inhibition, the IC50 values were 82.97 μg/mL (methanol), 202.70 μg/mL (n-hexane), and 97.04 μg/mL (diclofenac). Additionally, the n-hexane extract surpassed the methanol extract and diclofenac in BSA (bovine serum albumin) denaturation inhibition (90.4% vs. 22.0% and 51.4% at 62.5 μg/mL). The BSA denaturation IC50 values were 14.30 μg/mL (n-hexane), 5408 μg/mL (methanol), and 42.30 μg/mL (diclofenac). Gas chromatography–mass spectrometry (GC–MS) revealed 59 and 58 secondary metabolites in the methanol and n-hexane extracts, respectively. The higher therapeutic activity of the methanol extract was attributed to hydroxyacetic acid hydrazide, absent in the n-hexane extract. In silico docking studies identified 28 compounds with negative binding energies, indicating potential trypsin inhibition. The 2-hydroxyacetohydrazide displayed superior inhibitory effects compared to diclofenac. Further mechanistic studies are crucial to validate 2-hydroxyacetohydrazide as a potential drug candidate for rheumatoid arthritis treatment.

## 1. Introduction

Rheumatoid arthritis (RA) is an autoimmune disorder characterized by inflammatory changes in joint synovial tissue, cartilage, bone, and, occasionally, extra-articular locations [1]. The interaction between synovial-like fibroblasts, macrophages, and infiltrating lymphocytes leads to the production of inflammatory cytokines [2]. The inflammatory microenvironment can disrupt the balance between bone resorption by osteoclasts and bone production by osteoblasts, contributing to bone deterioration [3]. Various factors, such as ageing, smoking, immunogenicity, and genetic polymorphisms, may influence RA pathophysiology and contribute to its refractory nature [4]. Currently, non-steroidal anti-inflammatory drugs, immunosuppressive glucocorticoids, and disease-modifying antirheumatic drugs are used to alleviate RA symptoms and slow disease progression [5]. These drugs have been associated with important side effects, including gastrointestinal problems, hepatotoxicity, hepatitis, pneumonitis, and cardiovascular events [6]. An RA disability rate of 43–48% and a disease progression time of 5–10 years underscore the need for finding effective and safe treatments [4]. Plant-based therapeutics offer promising research and development opportunities because many of their active ingredients (e.g., triterpenoids, phenols, flavonoids, and polysaccharides) exhibit anti-inflammatory, analgesic, and immunomodulatory activities [7].

Medicinal plants for RA treatment have gained attention due to their potential therapeutic benefits and fewer side effects compared to conventional drugs [8]. Various plants with anti-inflammatory, analgesic, and immunomodulatory properties have been traditionally employed to alleviate RA symptoms. For instance, turmeric (*Curcuma longa*) contains the active compound curcumin, with anti-inflammatory effects [9]. Moreover, *Boswellia serrata*, commonly known as Indian frankincense, has recognized anti-inflammatory and analgesic properties [10]. Willow bark (*Salix* spp.) is another traditional remedy that contains salicin, a natural compound like aspirin with pain relief and anti-inflammatory effects [11]. The anti-arthritic potential of ginger (*Zingiber officinale*) has also been explored, and its active components exhibit anti-inflammatory and antioxidant properties [12]. Similarly, devil’s claw (*Harpagophytum procumbens*) and cat’s claw (*Uncaria tomentosa*) have been studied for managing RA symptoms [13]. However, more research and clinical studies are needed to establish their precise mechanisms and dosages for optimal therapeutic benefits in the context of RA treatment.

*Ammodaucus leucotrichus*, a plant within the Apiaceae family, stands out as a crucial element in Moroccan flora, with significant applications in traditional herbal medicine across North African nations. Known as ‘Kamune es sufi’ or ‘akâman’ in North African countries, and “Moudrayga” in Algeria, this glabrous annual plant thrives in the Saharan and sub-Saharan regions [14]. Its rich ethnobotanical uses include the treatment of various conditions, particularly noteworthy for its efficacy in addressing issues associated with RA. The plant has been traditionally employed to alleviate gastrointestinal problems, pulmonary diseases, labor pains, and various ailments in both children and adults [14]. Of particular relevance to RA treatment, *Ammodaucus leucotrichus* has demonstrated its effectiveness in managing conditions such as cystitis, nephritic colics, and kidney stones. Moreover, its role as a sugar regulator for diabetics further underscores its therapeutic potential [14]. The plant’s extracts, abundant in phytochemicals like perillaldehyde and limonene, exhibit a wide array of pharmacological activities, including anti-inflammatory effects [14]. This makes *Ammodaucus leucotrichus* a promising candidate for further research and development in the pharmaceutical industry, particularly in the quest for novel and effective treatments for rheumatoid arthritis.

This study addressed the limitations and side effects associated with the existing anti-inflammatory treatments for RA by focusing on innovative therapeutic strategies, with a particular emphasis on *Ammodaucus leucotrichus* extracts. This study explored the anti-inflammatory and anti-arthritic properties of *Ammodaucus leucotrichus* seed extracts obtained using methanol and n-hexane as solvents. The methodology involved comprehensive analyses, including gas chromatography–mass spectrometry (GC–MS), to identify the phytochemical constituents. In vitro assessments included protein denaturation assays using bovine serum albumin (BSA) and protease inhibition assays using trypsin, an inflammation enzyme. In silico molecular docking studies were used to assess the binding affinity of key compounds identified in the methanol extract for trypsin, an enzyme essential in RA-linked processes. Moreover, the interaction of the phytocompounds identified in the methanol extract with ten human enzymes was investigated by determining their binding affinity with the PASS tool, which was used to predict their biological activity, and ADME/T to predict their absorption, distribution, metabolism, excretion, and toxicity. This pivotal study explored the potential of *Ammodaucus leucotrichus* seed extracts for developing novel and safer treatments for RA, with the goal of overcoming the challenges associated with conventional drugs.

## 2. Results and Discussion

Seed extracts from *Ammodaucus leucotrichus* were investigated using methanol and n-hexane, focusing on anti-inflammatory and anti-arthritic properties. The methanol extract outperformed the n-hexane extract and diclofenac, a reference anti-inflammatory drug. The selection of solvents in our extraction process, namely methanol and n-hexane, was meticulously undertaken based on their distinct properties that allow for the selective extraction of specific classes of compounds from *Ammodaucus leucotrichus* seeds. Methanol, being a polar solvent, is proficient at extracting polar compounds such as phenolic compounds, flavonoids, and certain organic acids. On the other hand, n-hexane, being non-polar, excels in extracting lipophilic compounds like oils, fatty acids, and hydrophobic substances. The choice to avoid water as a solvent in the extraction of *Ammodaucus leucotrichus* seeds was driven by the nature of the target compounds. Water, being a polar solvent, might not effectively extract certain non-polar compounds present in the seeds, potentially leading to incomplete representation of the seed’s chemical composition. In contrast, the combination of methanol and n-hexane offers a comprehensive extraction approach, ensuring that a broader spectrum of compounds are captured in our analysis.

### 2.1. GC–MS Analysis of the Extracts

GC–MS analysis found 59 and 58 therapeutic secondary metabolites in the methanol and n-hexane extracts. Validation included peak area, molecular weight, retention time, PubChem CID number, and chemical formula. Details of the GC–MS analysis are summarized in Tables S1–S3 and Figure S1 (Supplementary Materials). These secondary metabolites were classified into different biological and pharmacological categories, such as ketones, amino alcohols, hydrazide derivatives, pyranone derivatives, furfural derivatives, aldehydes, phenol derivatives, sulfonate esters, cycloalkanols, carboxylic acids, tricyclic sesquiterpenes, sesquiterpene alcohols, heterocyclic compounds, steroids, spiro compounds, organosilicon compounds, tricyclic alcohols, phenone oximes, bicyclic terpenes, terpene alcohols, phthalate esters, siloxanes, sugar derivatives, nitrobenzofurans, cyclopropane derivatives, fatty acids, saturated hydrocarbons, and terpene esters.

Table S1 (Supplementary Materials) presents phytochemicals in methanol extract of *Ammodaucus leucotrichus* seed, highlighting the diverse chemical classes found in natural plant products. Major classes include fatty acids, terpenes, and mycotoxins [14]. Fatty acids play vital roles in biological processes, serving as lipid and signaling molecule components [15]. Terpenes, such as neophytadiene and isospathulenol, contribute to plant flavors and exhibit medicinal properties. The presence of trichothecenes suggests the potential existence of mycotoxins, known for their toxicity and potential medicinal uses [16]. This chemical diversity reflects the plant’s adaptation and defense mechanisms. The compounds offer potential biological activities, with the effects in RA depending on concentration, bioavailability, and interactions. Fatty acids may display anti-inflammatory properties, potentially alleviating RA symptoms [15]. Terpenes, including isospathulenol, studied for their anti-inflammatory and antioxidant effects, might aid in managing RA inflammation [17]. Incorporating docking studies could enhance understanding by investigating the interactions between these compounds and specific targets implicated in RA, providing valuable insights for future research and therapeutic development.

Table S2 (Supplementary Materials) presents phytochemicals in n-hexane extract of *Ammodaucus leucotrichus* seeds, highlighting the diverse chemical classes found in natural plant products. The n-hexane extract of *Ammodaucus leucotrichus* seeds contains a diverse array of compounds with potential implications for therapeutic applications. Notably, fatty acids such as 9-Octadecenoic acid (Octadec-9-enoic acid) and Octadeca-9,12-dienoic acid exhibit characteristics commonly associated with anti-inflammatory effects. Terpenoids like 3-Isopropyl-6,7-dimethyltricyclo [4.4.0.(2,8)]decane-9,10-diol and spathulenol have demonstrated anti-inflammatory and antioxidant properties, potentially contributing to the management of conditions like RA [18]. The presence of alcohols, esters, and hydrocarbons further diversifies the extract, highlighting the complex nature of the plant’s chemical composition. Molecular docking studies could shed light on the specific interactions between these compounds and molecular targets relevant to RA, potentially uncovering novel therapeutic avenues. 

The differences in chemical structure and solvation power between the solvents n-hexane and methanol are explained by their distinct polarities [19,20]. The differences are evident in the peak area percentages of various compound classes for both extracts (Table 1). Notably, some compound classes that may have an effect on rheumatoid arthritis (e.g., amino alcohols, hydrazide derivatives, pyranone derivatives, furfural derivatives, sulfonate esters, cycloalkanols, tricyclic sesquiterpenes, heterocyclic compounds, steroids, organosilicon compounds, phenone oximes, bicyclic terpenes, phthalate esters, siloxanes, sugar derivatives, nitrobenzofurans, cyclopropane derivatives, and terpene esters) were absent in the n-hexane extract. In this class, hydrazide derivatives, heterocyclic compounds, and phenone oximes showed more significant effects in rheumatoid arthritis compared to diclofenac, as tested in silico afterward.

Analysis of the results in Table 1 showed that the methanol extract included mainly fatty acids (62.90%), followed by terpene alcohols (10.59%) and terpene esters (2.48%). The n-hexane extract also included mainly fatty acids (68.09%), lower percentages of tricyclic alcohols (3.65%), and terpene alcohols (0.11%). Moreover, n-hexadecanoic acid, with known anti-inflammatory effects, was identified in both extracts. Previous studies suggest that n-hexadecanoic acid has anti-inflammatory effects by inhibiting various inflammatory mediators, including phospholipase A2, prostaglandins E2, interleukin (IL)-6, IL-1, tumor necrosis factor, and nitric oxide synthase. Additionally, it exhibits hypocholesterolemic, nematicidal, and pesticidal effects [21,22,23,24,25,26]. The fatty acids identified are in line with the findings from a previous study [27]. Another noteworthy compound was 4-Prop-1-en-2-ylcyclohexene-1-carbaldehyde, commonly known as perillaldehyde. This chemical (molecular formula: C10H14O; molecular weight: 150.22 g/mol) was newly identified in both extracts and had a peak area of 0.35% in the methanol extract and 3.02% in the n-hexane extract.

A review by Idm’hand, Msanda, and Cherifi [14] offered comprehensive insights into the phytochemistry and pharmacological attributes of *Ammodaucus leucotrichus*. Their thorough analysis revealed an extensive phytochemical composition, comprising 129 compounds from diverse chemical classes, including terpenoids, aldehydes, alcohols, ketones, and other bioactive constituents. This array of compounds suggested broad pharmacological potential and versatile bioactive properties for potential therapeutic applications. The study detailed the phytochemicals isolated from various plant parts, referencing seeds, fruits, aerial parts, and others. The identified compounds ranged from well-known substances like limonene, perillaldehyde, and α-pinene to a variety of terpenoids, alcohols, and ketones. In contrast, our study took an experimental approach, focusing specifically on the anti-inflammatory and anti-arthritic properties of *Ammodaucus leucotrichus* seed extracts obtained using methanol and n-hexane as solvents. This targeted experimental work aimed to explore the potential therapeutic benefits of the plant’s seed extracts in the context of RA.

**Table 1:** Comparative analysis of phytochemicals in the methanol and n-hexane extracts: peak area percentages from the GC–MS (gas chromatography–mass spectrometry) analysis and therapeutic effect on RA based on previous studies.

Class	Methanol Extract	Peak Area (%)	n-Hexane Extract	Peak Area (%)	Anti-RA Effect
Fatty acids and derivatives	Tetradecanoic acid;Methyl (9Z,12Z)-octadeca-9,12-dienoate; (9Z, 12Z)-Octadeca-9,12-dienoic acid; 9-octadecenoic acid; octadecanoic acid; Methyl hexadecanoate; n-hexadecanoic acid; heptadecanoic acid; Methyl (9E)-octadec-9-enoate	62.90	Methyl (9Z,12Z)-octadeca-9,12-dienoate; (9Z, 12Z)-Octadeca-9,12-dienoic acid; 9-octadecenoic acid; octadecanoic acid; Methyl hexadecanoate; n-hexadecanoic acid; Methyl (9E)-octadec-9-enoate	68.09	Yes [28,29]
Terpene alcohols	2,4,7,14-Tetramethyl-4-vinyl-tricyclo [5.4.3.0(1,8)]tetradecan-6-ol; bicyclo [4.4.0]dec-2-ene-4-ol, 2-methyl-9-(prop-1-en-3-ol-2)-	10.59	(S)-(-)-(4-isopropenyl-1-cyclohexenyl)methanol	0.11	Yes [30]
Terpene esters	Trans-(R,R)-chrysanthemyl (R)-2-methylbutanoate; kauren-19-yl-acetate; Methyl-5,9,13-trimethyltetradecanoate	4.23	Not present	0.00	Not tested
Phthalate esters	Phthalic acid, tetradecyl trans-dec-3-enyl ester; phthalic acid, butyl hept-4-yl ester	2.42	Not present	0.00	Not tested
Sesquiterpene alcohols	Isospathulenol, thunbergol, 1-(1,5-Dimethylhexyl)-10-hydroxy-3a,6,6,9a,11a-pentamethylhexadecahydrocyclopenta [7,8]- phenanthro [8a,9-b]oxiren-7-yl acetate, (7S)-1,1,7-Trimethyl-4-methylidene-1α,2,3,4α,5,6,7α,7β-octahydrocyclopropa[h]azulen-7-ol; methyl 16-R/S-hydroxy-cleroda-3,13(14)-Z-dien-15,16-olide	2.39	Isospathulenol, (7S)-1,1,7-Trimethyl-4-methylidene-1α,2,3,4α,5,6,7α,7β-octahydrocyclopropa[h]azulen-7-o, methyl 16-R/S-hydroxy-cleroda-3,13(14)-Z-dien-15,16-olide; (1R,7S,E)-7-isopropyl-4,10-dimethylenecyclodec-5-enol	2.72	Not tested
Hydrazide derivatives	2-hydroxyacetohydrazide; 3-hydroxy-3-methyl-butyric acid, hydrazide	1.91	Not present	0.00	Yes [31]
Bicyclic terpenes	2-Ethylidene-1,7,7-trimethylbicyclo [2.2.1]heptane; 8-isopropyl-1,5-dimethyltricyclo [4.4.0.02,7]dec-4-en-3-one	1.08	Not present	0.00	Not tested
Ketones	4,4-Dimethylpentan-2-one	1.36	4-Isopropenylcyclohexanone	0.46	Yes [20,32,33]
Tricyclic alcohols	3-Isopropyl-6,7-dimethyltricyclo [4.4.0.0(2,8)]decane-9,10-diol	0.97	3-Isopropyl-6,7-dimethyltricyclo [4.4.0.0(2,8)]decane-9,10-diol	3.65	Yes [34]
Furfural derivatives	5-Hydroxymethylfurfural	0.87	Not present	0.00	Yes [32]
Cyclopropane derivatives	Cyclopropanebutanoic acid, Methyl-4-[2-[[2-[[2-[(2-pentylcyclopropyl)methyl]cyclopropyl]methyl]cyclopropyl]methyl]-cyclopropyl]butanoate	0.71	Not present	0.00	Not tested
Saturated hydrocarbons	Tetracontane	0.68	3-Methylheptane; 3-Methylhexane; 2,2-Dimethylhexane; heptane	1.68	Not tested
Nitrobenzofurans	5-nitrobenzofuran-2(3H)-one	0.64	Not present	0.00	Not tested
Amino alcohols	(2R/2S)-2-Aminopropan-1-ol	0.57	Not present	0.00	Yes [35,36]
Tricyclic sesquiterpenes	(1R,4R,6R,10S)-4,12,12-Trimethyl-9-methylene-5-oxatricyclo [8.2.0.04,6]dodecane	0.55	Not present	0.00	Yes [37]
Carboxylic acids and derivatives	4-Prop-1-en-2-ylcyclohexene-1-carbaldehyde; Undecyl methanoate	0.48	Dodecanoic acid, 2-(Tricyclo [3.3.1.13,7]dec-1-yl)propanoic acid	0.40	Yes [38,39,40]
Spiro compounds	Spiro [5.6]dodecan-7-one, spiro [5.5]undeca-1,7-diene	0.45	5,5-Diethyl-4-methyl-6-spiro [2.3] hexane	1.05	Not tested
Steroids	3beta-trimethylsiloxy-5alpha,6alpha-epoxycholestane	0.43	Not present	0.00	Not tested
Heterocyclic compounds	2,2-bis(oxidanylidene)-1,5-dihydroimidazo [4,5-c][1,2,6]thiadiazin-4-one	0.38	Not present	0.00	Not tested
Organosilicon compounds	Trichloro(dodecyl)silane	0.38	Not present	0.00	Not tested
Aldehydes	4-Prop-1-en-2-ylcyclohexene-1-carboxylic acid	0.35	4-Prop-1-en-2-ylcyclohexene-1-carboxylic acid	3.07	Not tested
Siloxanes	Octadecamethyl-cyclononasiloxane	0.33	Not present	0.00	Not tested
Sugar derivatives	1,2,3,4,5-Penta-O-acetyl-D-xylitol	0.27	Not present	0.00	Not tested
Phenol derivatives	Thymol	0.25	2,6-Dimethoxy-4-(prop-2-en-1-yl)phenol	0.79	Yes [41]
Phenone oximes	4’-Hydroxybutyrophenone oxime	0.22	Not present	0.00	Yes [42]
Pyranone derivatives	3,5-Dihydroxy-6-methyl-2,3-dihydropyran-4-one	0.20	Not present	0.00	Yes [32]
Cycloalkanols	Cyclopentanol	0.19	Not present	0.00	Not tested
Sulfonate esters	[(Z)-4-Methylsulfonyloxybut-2-enyl] 2-(tert-butoxycarbonylamino)acetate	0.13	Not present	0.00	Yes [33]

### 2.2. Protein Denaturation Assay

**Figure 1:**
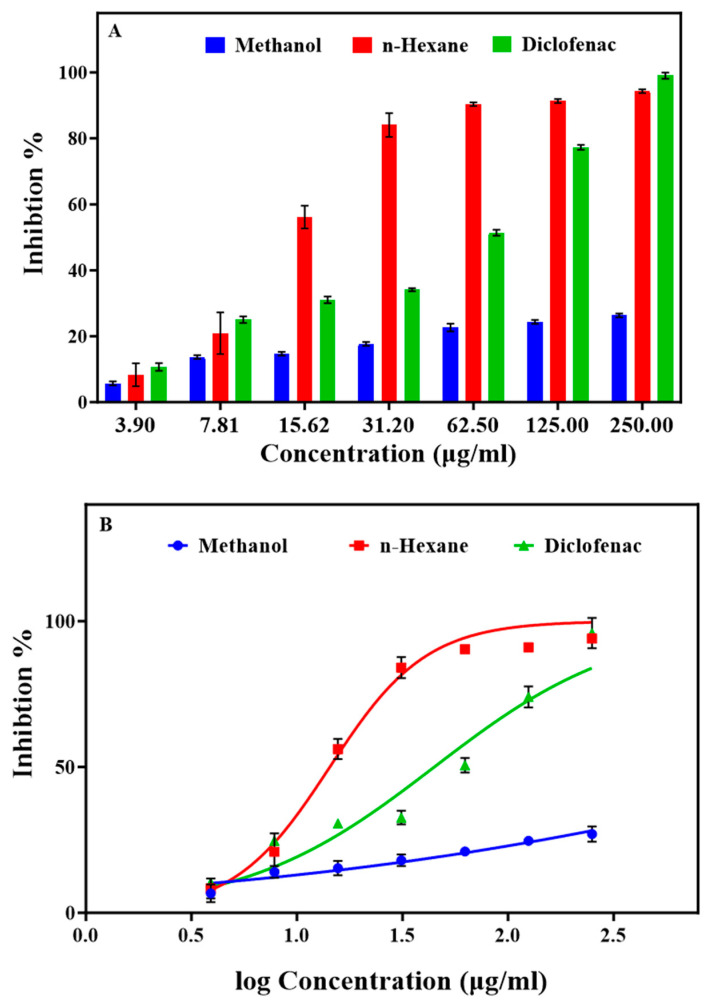
Effect of different concentrations of the *Ammodaucus leucotrichus* seed methanol and n-hexane extracts on BSA denaturation. (**A**) Comparison of the extracts with diclofenac in a protein denaturation assay and (**B**) IC_50_ values (μg/mL) for BSA denaturation inhibition. Data are the mean ± standard error of the mean.

The anti-inflammatory properties of methanol and n-hexane extracts were assessed using in vitro protein denaturation and protease activity (crucial factors in chronic inflammatory conditions like RA) inhibition assays. Protein denaturation, which is associated with inflammation, was evaluated using a BSA denaturation assay. The strongest BSA denaturation inhibition was obtained with the n-hexane extract at 62.5 µg/mL: 90.36% vs. 51.36% for diclofenac (the reference drug) at the same concentration (Figure 1A). Conversely, the methanol extract exhibited a modest effect, reaching an IC_50_ of 5408.00 µg/mL, compared with the n-hexane extract (IC_50_ = 14.30 µg/mL) and also diclofenac (IC_50_ = 42.30 µg/mL) (Figure 1 and Table S4). This emphasizes the remarkable effectiveness of the n-hexane extract in inhibiting BSA denaturation compared with the methanol extract and also diclofenac, suggesting its potential to inhibit the release of lysosomal material by neutrophils at the inflammation site [43,44].

### 2.3. Protease Inhibition Activity

**Figure 2:**
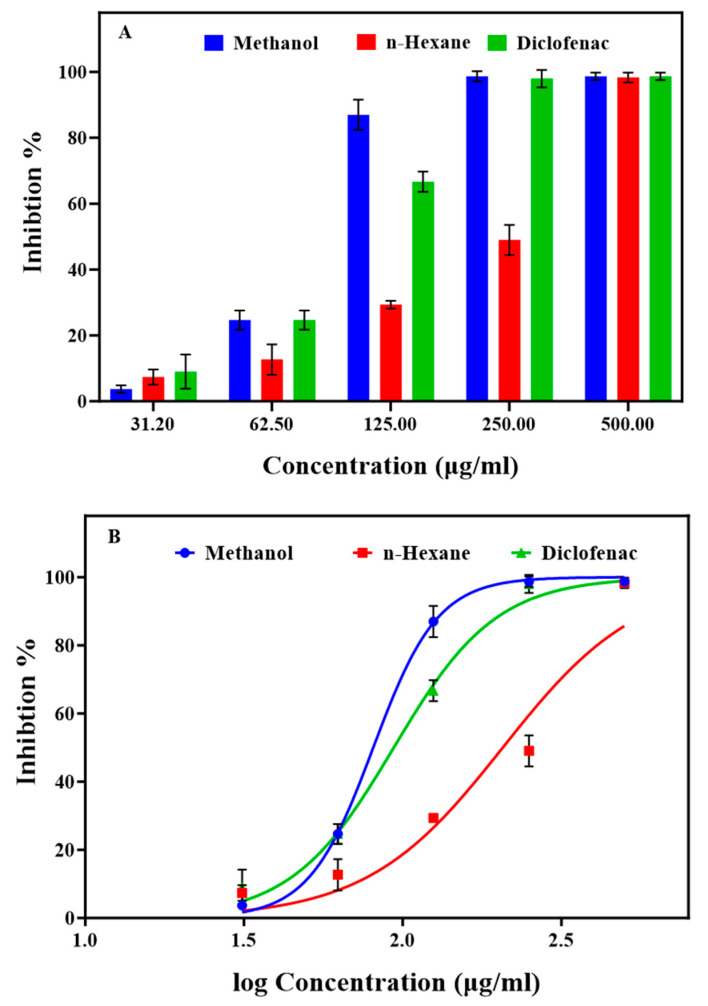
Influence of various concentrations of the methanol and n-hexane extracts from *Ammodaucus leucotrichus* seeds on protease (trypsin) inhibition. (**A**) Percentage of trypsin inhibition compared with diclofenac, the reference drug, and (**B**) IC_50_ values (μg/mL) for trypsin inhibition. Data are the mean ± standard error of the mean.

Figure 2A illustrates the significant trypsin inhibition demonstrated by both extracts at various concentrations, implying their potential for RA treatment (Table S5 in the Supplementary Materials). The methanol extracts, along with diclofenac, achieved complete inhibition (100%) at a concentration of 250 μg/mL. The n-hexane extract exhibited lower (51%), but still significant inhibition, at the same concentration. The IC50 values (Figure 2B and Table S5) were 82.97 μg/mL for the methanol extract, 97.04 μg/mL for diclofenac, and 202.7 μg/mL for the n-hexane extract. The high trypsin inhibition by the methanol extract suggests considerable anti-inflammatory effects, as reported by Biswajita, P et al. [45] and Mane, M. P et al. [46] using other plant extracts (Enteromorpha intestinalis and Polygala arvensis). Protease inhibitors have demonstrated efficacy in various clinical diseases, including cancer, AIDS, RA, pancreatitis, and thrombosis [47]. Therefore, the methanol extract holds promise for RA by effectively inhibiting proteases, offering a natural alternative approach to diclofenac, a non-steroidal anti-inflammatory drug with important side effects.

### 2.4. Molecular Docking

Earlier assays clearly demonstrated the exclusive anti-protease efficacy of the methanol extract, specifically against trypsin. To elucidate the responsible compounds, an in silico screening of the 59 identified phytochemicals within the methanol extract was conducted, utilizing drug design tools crucial for advancing drug development [48]. Molecular docking simulations with trypsin unveiled a diverse range of affinities among the 59 key compounds, with the hydrazide group, including 2-hydroxyacetohydrazide and 3-Hydroxy-3-methyl-butyric acid hydrazide (S1 and S2 in Table S6), standing out for its notable inhibitory potential against RA. The substantial binding energies of these hydrazides highlight their promising candidacy for further exploration in RA inhibition.

**Figure 3:**
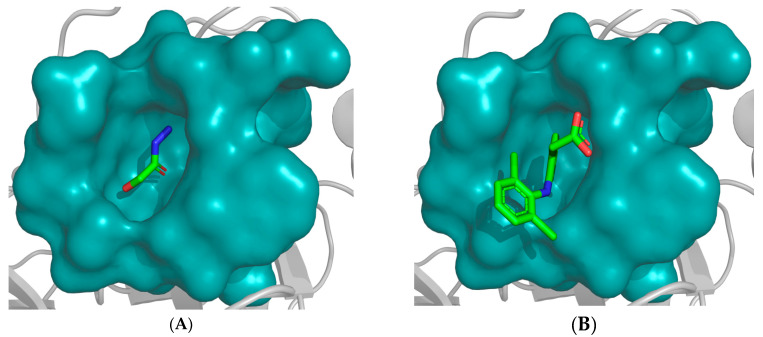
Placement of (**A**) 2-hydroxyacetohydrazide and (**B**) diclofenac within trypsin active site. The most plausible conformation of each compound, obtained by FlexX, is illustrated. The active site is represented in cyan as a ‘surface mode’, with the ligand atoms color-coded: carbon in green, oxygen in red, and nitrogen in blue.

**Figure 4:**
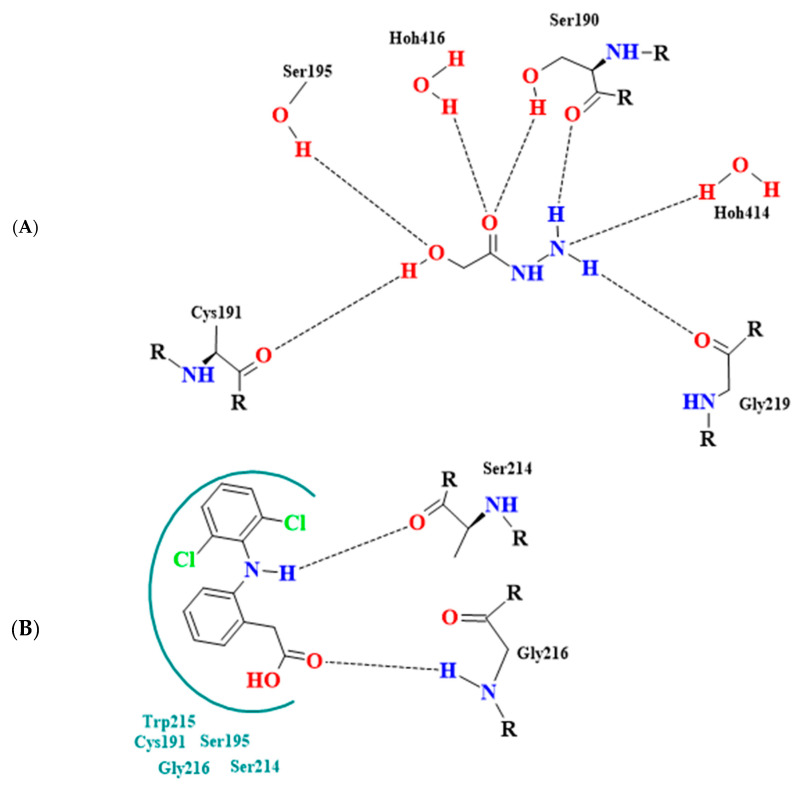
Binding mode interactions of (**A**) 2-hydroxyacetohydrazide from the extract and (**B**) diclofenac (reference drug) within trypsin active site (inflammation-related enzyme); hydrogen bonds depicted by broken lines.

Notably, 2-hydroxyacetohydrazide exhibited a more negative binding energy than diclofenac, emphasizing its inhibitory potency. Visual analysis underscores the rational orientation of 2-hydroxyacetohydrazide and diclofenac within the trypsin active site (Figure 3). Remarkably, 2-hydroxyacetohydrazide formed seven hydrogen bonds with crucial residues (Cys191, Ser195, Ser190, and Gly219), indicating a robust inhibitory potential. In contrast, diclofenac formed only two hydrogen bonds (Figure 4) [48]. The predictive analysis unveiled multi-faceted actions, encompassing sphinganine kinase inhibition, G-protein-coupled receptor kinase inhibition, JAK2 expression inhibition, NF-kB activation, immunosuppressant effects, and complement factor D inhibition. These insights provide a comprehensive foundation for guiding future experimental investigations into the therapeutic potential of *Ammodaucus leucotrichus* seed extract, positioning it as a promising source for developing effective anti-RA agents.

Furthermore, the maintenance of bone and cartilage integrity crucially depends on balanced proteolytic activity, and various enzymes contribute to pro-inflammatory functions in RA [49]. Serine proteases, such as trypsin, play a role in the complement cascade, extending beyond the traditional roles of human collagenases in collagen breakdown [50]. The molecular docking results accentuate that 2-hydroxyacetohydrazide from *Ammodaucus leucotrichus* exhibits tighter binding to the target protein trypsin than diclofenac. This underscores 2-hydroxyacetohydrazide as a promising candidate for future RA research, showcasing its potential in modulating proteolytic activity associated with RA pathology.

### 2.5. Prediction of Potential Protein Targets and Druglikeness Analysis

**Figure 5:**
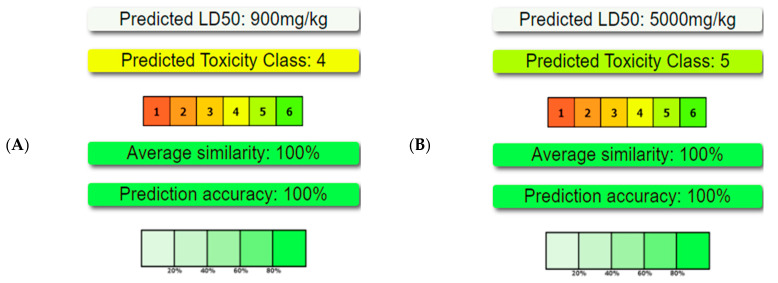

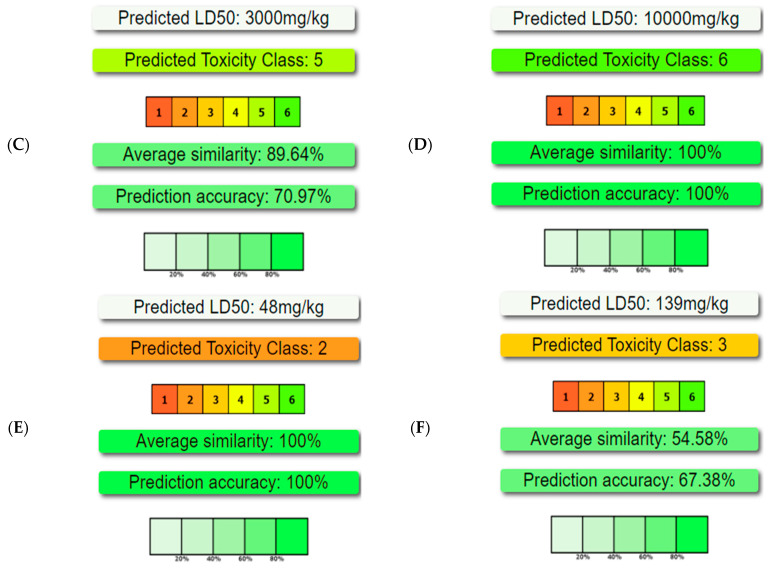
Toxicity, calculated by ProTox-II, of key compounds identified in the *Ammodaucus leucotrichus* seed methanol extract. The compounds include (**A**) n-hexadecanoic acid, (**B**) hexadecanoic acid methyl ester, (**C**) 9-octadecenoic acid methyl ester (E)-, (**D**) (9Z, 12Z)-Octadeca-9,12-dienoic acid, (**E**) 9-octadecenoic acid, and (**F**) 2-hydroxyacetohydrazide.

The screening of the 59 phytochemicals from the methanol extract of *Ammodaucus leucotrichus* seeds also involved using the online PASS tool to predict their biological activity spectrum (Figure 5). This tool provides insights into the potential pharmacological effects, mechanisms of action, and toxicity based on the compound chemical structure [51]. The diversity of predicted activities encompassed anti-inflammatory effects and targeting of key molecules associated with RA. The tool successfully identified 2-hydroxyacetohydrazide in Table S7 (Supplementary Materials) as a potent agent with anti-inflammatory effects (Pa: 0.004) and sphinganine kinase inhibition activity. This positions 2-hydroxyacetohydrazide as a promising multi-faceted candidate for RA treatment. Notably, the tool encountered challenges predicting outcomes for 2-hydroxyacetohydrazide, possibly attributable to its relatively short carbon chain.

The identified phytochemicals from the methanol extract of *Ammodaucus leucotrichus* seeds target various key pathways associated with RA. Sphinganine kinase, a crucial enzyme in the regulation of inflammatory factors and immune responses, was targeted by several compounds, including 2-hydroxyacetohydrazide, emphasizing their potential in modulating sphingosine metabolism [52,53]. G-protein-coupled receptors, implicated in RA mechanisms, were also affected by multiple compounds, suggesting a role in modulating receptor signaling for therapeutic benefits [54,55,56]. Inhibition of Janus kinases (JAKs) by certain compounds aligns with the emerging class of JAK inhibitors in RA treatment, demonstrating potential efficacy in disease modification [57,58]. Compounds displaying immunosuppressant properties hold promise for mitigating chronic inflammation and pain associated with RA [59,60]. Furthermore, the prediction of matrix metalloproteinase (MMP) inhibition, particularly MMP9, suggests a potential avenue for joint protection in RA [61,62]. Transcription factor NF-κB, a key regulator of inflammation, was targeted by several compounds, providing insights into potential anti-inflammatory effects [63,64]. Notably, the hydrazide of 2-hydroxyacetohydrazide stands out, possibly due to its unique structure.

Six selected compounds, including 2-hydroxyacetohydrazide, n-hexadecanoic acid, hexadecanoic acid methyl ester, (9Z,12Z)-Octadeca-9,12-dienoic acid, and (E) 9-octadecenoic acid, demonstrated promising effects on multiple RA therapeutic targets. This selection process, involving in vitro and in silico assessments along with PASS predictions (Figure 5), underscores a comprehensive approach to identifying potential anti-RA candidates [65]. To further evaluate these compounds, pharmacokinetic properties and toxicity were assessed. SwissADME and ProTox-II results indicated low toxicity for n-hexadecanoic acid, hexadecanoic acid methyl ester, (9Z,12Z)-Octadeca-9,12-dienoic acid, and (E) 9-octadecenoic acid, while 9-octadecenoic acid and 2-hydroxyacetohydrazide exhibited higher toxicity, highlighting the importance of considering safety profiles in drug development [66]. Additionally, Swiss Target Prediction revealed potential interactions with enzymes, fatty-acid-binding proteins, G-protein-coupled receptors, and nuclear receptors, providing valuable insights into the biological activity of these compounds [65]. Overall, this comprehensive analysis offers a foundation for further experimental exploration and potential drug development for RA treatment.

**Figure 6:**
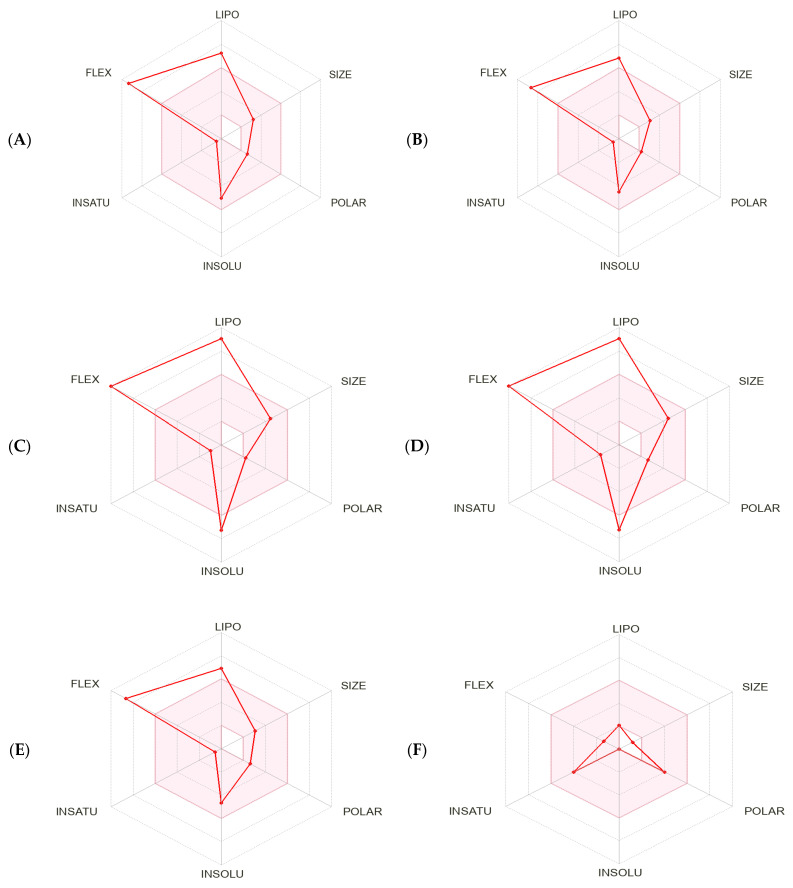

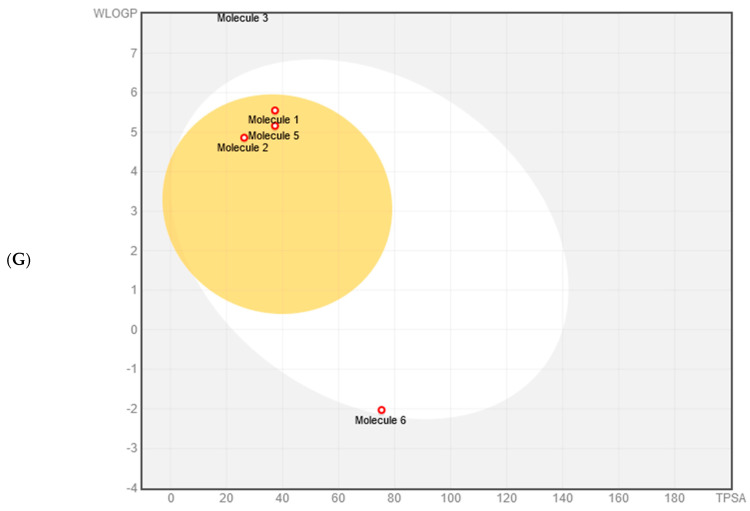
Bioavailability radar charts within the ADME property domain borders, calculated by SwissADME, for the key compounds identified in the methanol extract of *Ammodaucus leucotrichus* seeds: (**A**) n-hexadecanoic acid, (**B**) hexadecanoic acid methyl ester, (**C**) 9-octadecenoic acid methyl ester (E)-, (**D**) (9Z, 12Z)-Octadeca-9,12-dienoic acid, (**E**) 9-octadecenoic acid, and (**F**) 2-hydroxyacetohydrazide. (**G**) BOILED-Egg model. The pink area in the radar chart shows the optimal range for the following features: lipophilicity (LIPO), size, polarity (POLAR), solubility (INSOLU), saturation (INSATU), and flexibility (FLEX).

The bioavailability radar charts generated by SwissADME [66] stressed the favorable characteristics of the compounds: high scores for size, polarity, solubility, and saturation, and low scores for flexibility and lipophilicity. Moreover, 2-hydroxyacetohydrazide was within the optimal range for all the tested characteristics (Figure 6 and Table 2). The BOILED-Egg method was used to predict blood–brain barrier (BBB) access and passive gastrointestinal absorption. Key compounds (n-hexadecanoic acid, hexadecanoic acid methyl ester, and 9-octadecenoic acid) were inside the yolk, suggesting high BBB permeation. Only 2-hydroxyacetohydrazide was in the white part, suggesting favorable gastrointestinal absorption due to its lower WLOGP value (a lipophilicity indicator) and higher Total Polar Surface Area (TPSA) value compared with the other compounds. The outer gray region indicates molecules with low absorption and limited BBB penetration: 9-octadecenoic acid methyl ester (E)- and (9Z, 12Z)-Octadeca-9,12-dienoic acid. The SwissADME results suggest a minimal impact of P-glycoproteins (P-gp) in the central nervous system on the main substances present in the methanol extract, as indicated by the red dots. SwissADME also predicted that most of the compounds but n-hexadecanoic acid and 9-octadecenoic acid should not inhibit major cytochrome P450 (CYP) isoforms (CYP2C19, CYP2D6, CYP3A4, and CYP2C9). Conversely, all the tested compounds but 2-hydroxyacetohydrazide should inhibit CYP1A2 (Table 2). The physicochemical properties, crucial for efficacy, safety, and metabolism, were assessed using six rule-based methods, including Lipinski’s Rule of Five (RO5), confirming full compliance, with molecular mass <500 daltons, hydrogen bond donors (HBD) <5, hydrogen bond acceptors (HBA) <10, and octanol–water partition coefficient (Clog *P*) ≤5 for all the selected molecules (Table 2).

Table 2 provides the comprehensive in silico ADMET profile of the six key compounds identified in the methanol extract of *Ammodaucus leucotrichus* seeds. The TPSA values ranged from 26.30 to 75.35 Å², and 2-hydroxyacetohydrazide had the highest TPSA. The consensus log Po/w, an indicator of lipophilicity, varied between 5.20 and −1.51; n-hexadecanoic acid displayed the highest lipophilicity and 2-hydroxyacetohydrazide was hydrophilic. All the compounds were predicted to have substantial gastrointestinal absorption and complied with Lipinski’s Rule of Five, indicating their druglike properties. BBB penetration was predicted for n-hexadecanoic acid and hexadecanoic acid methyl ester but not for 2-hydroxyacetohydrazide. Importantly, the lack of P-gp substrate prediction suggests favorable bioavailability. Although some compounds were predicted to inhibit specific CYP isoforms, the majority of them displayed interesting characteristics for effective drug development and therapeutic applications, underscoring their potential as promising drug candidates that deserve further exploration and experimental validation.

## 3. Materials and Methods 

### 3.1. Phytochemical Extraction

*Ammodaucus leucotrichus* seeds were collected in the Bechar region (part of the arid Algerian Sahara) in the southwest of Algeria during the summer of 2021. Professor Sabah Charmat from Ferhat Abbas University Setif 1, Algeria, confirmed their authenticity. An official voucher specimen was documented and assigned the reference number 220/SNV/DA/UFAS/21. The seed powder underwent solvent extraction using absolute methanol (CH_3_OH, ≥99.8%, Sigma-Aldrich, Burlington, MA, USA) or n-hexane (CH_3_(CH2)_4_CH_3_, ≥97.0%, Sigma-Aldrich) following the method outlined by Arrar et al. [67]. Briefly, 5 kg of seed powder was immersed in absolute methanol or n-hexane in a sealed container and left at room temperature (27 °C) with regular agitation for 3–7 days until complete dissolution of the soluble components. The solutions were filtered through Whatman No. 4.1 filter paper and concentrated under reduced pressure using a rotary evaporator at 40 °C, yielding the crude extracts, dissolved in distilled water and kept at 4 °C.

### 3.2. Phytochemical Analysis by GC–MS

GC–MS (gas chromatography–mass spectrometry) analysis was completed using a Hewlett-Packard 6890 system interfaced with a quadrupole mass spectrometer (model HP 5973), equipped with an HP5 MS capillary column (5% phenylmethyl siloxane in dimethylpolysiloxane, 30 m × 0.25 mm, 0.25 mm film thickness; PTAPC, BISKRA). The experimental setup included helium carrier gas flow rate of 0.5 mL/min, split ratio of 30, electron ionization system with ionization energy of 70 eV, and scan range of 30–550 atomic mass units. The GC–MS transfer line temperatures for the injector and detector were set at 250 °C and 280 °C, respectively, and the ion source temperature was maintained at 230 °C. The column temperature was at 60 °C for 8 min and then was increased gradually to 280 °C (2 °C/min) and held isothermal for 30 min. Then, 0.2 mL of n-hexane (HPLC grade 95%, Fisher Chemical) and methanol solution (HPLC grade, ≥99.9%, Sigma-Aldrich) was injected. Mass spectra were compared with the computer libraries Wiley 7N, National Institute of Standards and Technology (NIST) 02, and NIST 98 (NIST11 and Wiley 8) for compound identification [27].

### 3.3. Protein Denaturation Assay

The in vitro anti-inflammatory effects of methanol and n-hexane extracts were examined using a protein denaturation assay, based on BSA (bovine serum albumin) (pH 7, ≥98%, Sigma-Aldrich), as reported by Suresh, P [68]. Briefly, 0.2% BSA in Tris-HCl buffer (pH 6.8, Trizma^®^ hydrochloride, >99%, Sigma-Aldrich) was combined with various extract concentrations (250, 125, 62.50, 31.20, 15.62, and 7.81, 3 μg/mL) solubilized in distilled water or the reference anti-inflammatory drug (diclofenac, DICLAMID^®^, 25 mg/mL). Mixtures were sealed and incubated at 37 °C in an oven for 15 min, followed by heating in a water bath at 70 °C for 5 min. Turbidity absorbance was measured at 660 nm using a UV–vis spectrophotometer (VIS-7220G). The protein denaturation inhibition percentage was calculated using the formula (Equation (1)):

Protein denaturation inhibition (%) = (1 − At/Ac) × 100(1)

where At is the absorbance of the test sample, and Ac is the absorbance of the control.

### 3.4. Protease Inhibition Activity

The protease inhibition activity analysis was carried out following a modified version of the protocol described by Ahmad, S et al. [69]. The reaction mixture included 200 μL of extract at various concentrations (500, 250, 125, 62.5, and 31.2 μg/mL) solubilized in distilled water, 200 μL of 25 mM Tris-HCl buffer (Trizma^®^ hydrochloride, >99%, Sigma-Aldrich) at pH 7, and 12 μL of 0.6 mg trypsin (1000–2000 units/mg solid, Sigma-Aldrich). After incubation at 37 °C for 5 min, 200 μL of 0.8% *w*/*v* ovalbumin (Sigma-Aldrich) was added to the mixtures that were then incubated at 37 °C for 20 min. The reaction was stopped by adding 400 μL of 70% *v*/*v* hydrochloric acid (HCl, 37%, Sigma-Aldrich). This was followed by centrifugation (refrigerated centrifuge 3–30 KS, Sigma-Aldrich, Darmstadt, Germany) at 5000 rpm for 5 min to collect the supernatant, and absorbance was measured at 280 nm with a UV–vis spectrophotometer (VIS-7220G). Diclofenac served as reference standard. The percentage of protease inhibition percentage was calculated for each extract and diclofenac using the following formula (Equation (2)):

Protease inhibition (%) = (1 − At/Ac) × 100(2)

where At is the absorbance of the test sample, and Ac is the absorbance of the control.

### 3.5. In Silico Molecular Docking

To study the binding mechanisms of the compounds identified in the *Ammodaucus leucotrichus* seed methanol extract, a molecular docking study was conducted targeting the active sites of trypsin. The 3D coordinates for trypsin (ID: 2PTN) were obtained from the Protein Data Bank (https://www.rcsb.org, accessed on 5 Septembre 2023). Trypsin was prepared for docking using the LeadIT 2.1.8 software package (www.biosolveit.com, accessed on 5 Septembre 2023)). Before the docking simulation, all water molecules were removed, and polar hydrogen atoms were introduced [70]. Missing atoms were added, and formal charges were computed. Then, the resulting structure underwent rigorous minimization before being exported as mol2 files [71]. The 3D structure of each compound was retrieved from the PubChem database (https://pubchem.ncbi.nlm.nih.gov/, accessed on 05 Septembre 2023) and prepared for docking simulations using Maestro, version 11.3, and the LigPrep module (Schrödinger software company, New York, NY, USA) [72]. This module streamlines the generation of all tautomeric forms, protonation states at pH = 7.4 ± 1, and enantiomers for each ligand [73]. Molecular docking calculations were completed with FlexX 2.1.8 and an incremental ligand construction approach [74]. Fragment selection was set in automatic mode and the standard algorithm for fragment placement was used. Phytochemicals in the extract were ranked using the FlexX scoring function, providing scores in terms of changes in Gibbs free energy (ΔG, in kJ/mol).

### 3.6. Prediction of Potential Protein Targets and Druglikeness Analysis

To estimate the biological activity of the 59 compounds detected in the methanol extract, the PASS Online tool (way2drug.com/passonline/predict.php, accessed on 6 Septembre 2023) was employed (average accuracy >95%). The structures of the compounds were uploaded in “mol” format in PASS Online to obtain the probability to be active (Pa) and the probability to be inactive (Pi) values [75]. Then, the bioavailability and pharmacokinetic and toxicity properties of 2-hydroxyacetohydrazide were predicted using the SwissADME, BOILED-Egg, and ProTox-II—Prediction of toxicity of chemicals tools.

## 4. Conclusions

The evaluation of *Ammodaucus leucotrichus* seed extracts as potential anti-inflammatory and anti-arthritic agents involved employing methanol and n-hexane as solvents for the extraction of bioactive compounds. The extracts underwent assessment through protease (trypsin) and protein (BSA, bovine serum albumin) denaturation inhibition assays. The methanol extract demonstrated trypsin inhibition of 85%, surpassing both the n-hexane extract (30.0%) and diclofenac (64.67%) at 125 μg/mL. Conversely, the n-hexane extract exhibited the highest BSA denaturation inhibition rate at 90.4%, in comparison to the methanol extract (22.0%) and diclofenac (51.4%) at 62.5 μg/mL. GC–MS analysis revealed 59 and 58 secondary metabolites in the methanol and n-hexane extracts, respectively, indicating diverse bioactive compounds. In silico docking studies identified 28 compounds with negative binding energies, suggesting potential trypsin inhibition. Notably, 2-hydroxyacetohydrazide showed superior inhibitory effects (−17.13 Kj/mol) compared to diclofenac (−13.01 Kj/mol). The methanol extract, particularly 2-hydroxyacetohydrazide, emerged as a promising candidate for rheumatoid arthritis (RA) treatment due to potent trypsin inhibition. SwissADME analysis highlighted favorable bioavailability attributes, with optimal size, polarity, solubility, and saturation for 2-hydroxyacetohydrazide. The BOILED-Egg model predicted blood–brain barrier permeation and gastrointestinal absorption, providing insights into druglikeness and bioavailability. In summary, these findings propose the methanol extract as a promising candidate for anti-inflammatory applications, particularly in trypsin inhibition. However, we acknowledge the potential for alternative therapeutic approaches with the n-hexane extract, necessitating further investigation. The identified compound, 2-hydroxyacetohydrazide, shows promise as a potential drug candidate for rheumatoid arthritis treatment. Yet, rigorous mechanistic studies and validation are crucial to enhance our understanding of its therapeutic potential within the field of plant-based medicine.

## Figures and Tables

**Table 2:** In silico ADMET profiles of six phytocompounds identified in the methanol extract.

Entry	n-Hexadecanoic Acid	Hexadecenoic Acid, Methyl Ester	9-Octadecenoicacid, Methyl Ester, (E)-	9,12-Octadecadienoicacid(Z,Z)-	9-Octadecenoicacid	2-Hydroxyacetohydrazide
TPSA (Å²)	37.30	26.30	26.30	37.30	37.30	75.35
Consensus Log Po/w	5.20	5.54	5.95	5.95	5.71	−1.51
Gastrointestinal absorption	High	High	High	High	High	High
Bioavailability score	0.85	0.55	0.55	0.55	0.85	0.55
BBB access	Yes	Yes	No	No	No	No
P-gp substrate	No	No	No	No	No	No
CYP1A2	Yes	Yes	Yes	Yes	Yes	No
CYP2C19	No	No	No	No	No	No
CYP2C9	Yes	No	No	No	Yes	No
CYP2D6 CYP3A4	No	No	No	No	No	No
inhibitor	No	No	No	No	No	No
Lipinski	Yes	Yes	Yes	Yes	Yes	Yes

## Data Availability

Data is contained within the article and Supplementary Material.

## Supplementary Materials

The following supporting information can be downloaded at: https://www.mdpi.com/article/10.3390/ph17030385/s1, Figure S1. Gas chromatography-mass spectrometry analysis of (A) the methanol and (B) n-hexane extracts of *Ammodaucus leucotrichus* seeds. The chromatograms show the abundance of the different compounds in relation to their retention time (in minutes); Figure S2. Data on the predicted target of the selected compounds identified in the *Ammodaucus leucotrichus* methanol extract; Table S1. Comprehensive list of the phytochemicals identified in the methanol extract by gas chromatography-mass spectrometry. The table includes compound name, molecular formula, molecular weight, retention time, peak area, and PubChem Compound Identifier (CID); Table S2. Comprehensive list of the phytochemicals identified in the n-hexane extract by gas chromatography-mass spectrometry. The table includes compound name, molecular formula, molecular weight, retention time, peak area, and PubChem Compound Identifier (CID); Table S3. Comparison of the key phytocomponents (with their peak area) present in the methanol and n-hexane extracts, identified by gas chromatography/mass spectrometry; Table S4: Impact of different concentrations of the methanol and n-hexane extracts from *Ammodaucus leucotrichus* seeds on BSA denaturation. Comparison with diclofenac (reference anti-inflammatory drug) in a BSA denaturation assay and IC_50_ values (μg/ml) for BSA denaturation inhibition. Data are the mean ± standard error of the mean; Table S5: Trypsin inhibition by the methanol and n-hexane extracts from *Ammodaucus leucotrichus* seeds compared with Diclofenac (reference drug) at different concentrations, and IC_50_ values (μg/ml) for trypsin inhibition. Data are the mean ± standard error of the mean; Table S6: Binding energies (in kJ/mol) of the 59 key compounds identified in the methanol extract from *Ammodaucus leucotrichus* seeds and of diclofenac during their interactions with trypsin. The different binding energy values reflect variations in the inhibitory potential of the compounds against trypsin; Table S7. PASS prediction of the bioactivities of the 59 phytochemicals identified in the methanol extract from *Ammodaucus leucotrichus* seeds.
