# Supplementary material for: Exploring the Therapeutic Potential of Ammodaucus leucotrichus Seed Extracts: A Multi-Faceted Analysis of Phytochemical Composition, Anti-Inflammatory Efficacy, Predictive Anti-Arthritic Properties, and Molecular Docking Insights
Source: Pharmaceuticals (Basel). 2024 Mar 18;17(3):385. doi: 10.3390/ph17030385 (PMC10974001; doi:10.3390/ph17030385)
Supplement: Supplementary file 1 [file pharmaceuticals-17-00385-s001.zip › pharmaceuticals-2912164-supplementary.pdf]

pharmaceuticals

Article

Exploring the Therapeutic Potential of Ammodaucus leucotrichus Seed Extracts: A Multi-Faceted Analysis of Phytochemical Composition, Anti-Inflammatory Efficacy, Predictive Anti-Arthritic Properties, and Molecular Docking Insights

Cheima Djehiche [1], Nadia Benzidane [1], Hanene Djeghim [2], Mehdi Tebboub [3], El Hassen Mokrani [4], Saad Mebrek [2], Mohammed Messaoudi [5], Chawki Bensouici [2], Ali Alsalme [6], David Cornu [7], Mikhael Bechelany [7,8], Lekhmici Arrar [1,*] and Ahmed Barhoum [9,*]

[1] Laboratory of Applied Biochemistry, Department of Biochemistry, Faculty of Nature and Life Sciences, Ferhat Abbas University of Setif 1, Setif 19000, Algeria
[2] Biochemistry Laboratory, Division of Biotechnology and Health, Biotechnology Research Center (CRBt), Constantine 25000, Algeria
[3] Department of Mechanical Engineering, Faculty of Science and Technology, University Mentouri Brothers Constantine 1, Constantine 25000, Algeria
[4] Laboratory of Applied Biochemistry, Department of Biochemistry and Cellular and Molecular Biology, Faculty of Natural and Life Sciences, University Mentouri Brothers Constantine 1, Constantine 25000, Algeria
[5] Nuclear Research Centre of Birine, P.O. Box 180, Ain Oussera 17200, Algeria
[6] Department of Chemistry, College of Science, King Saud University, Riyadh 11451, Saudi Arabia
[7] Institut Européen des Membranes (IEM), UMR 5635, Univ. Montpellier, ENSCM, CNRS, Place Eugène Bataillon, 34095 Montpellier, France
[8] Functional Materials Group, Gulf University for Science and Technology (GUST), Mubarak Al-Abdullah 32093, Kuwait
[9] NanoStruc Research Group, Chemistry Department, Faculty of Science, Helwan University, Cairo 11795, Egypt
* Correspondence: lekharrar@univ-setif.dz (L.A.); ahmed.barhoum@science.helwan.edu.eg (A.B.)

Supplementary information

(A)

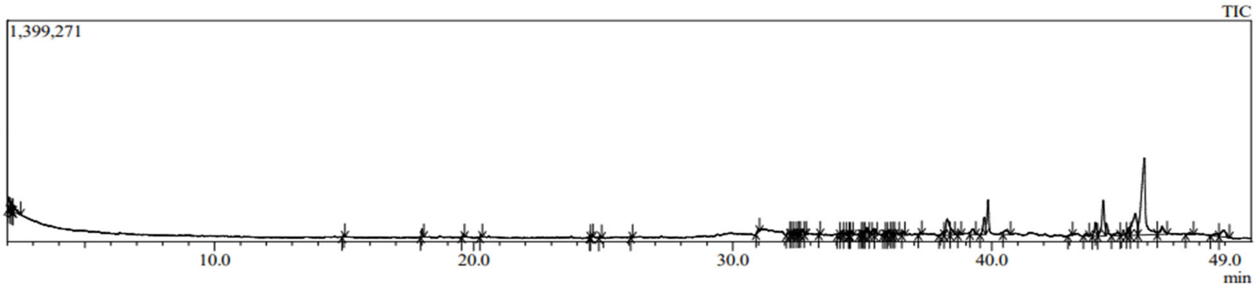

(B)

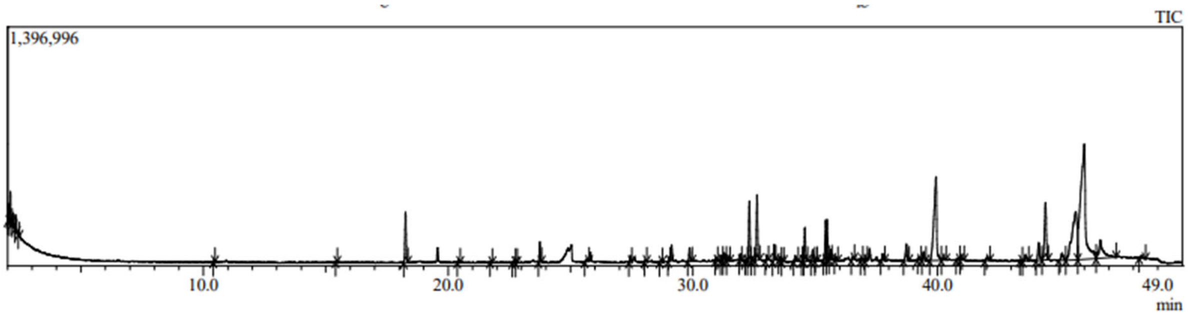

Figure S1. Gas chromatography-mass spectrometry analysis of (A) the methanol and (B) n-hexane extracts of *Ammodaucus leucotrichus* seeds. The chromatograms show the abundance of the different compounds in relation to their retention time (in minutes).

(A)

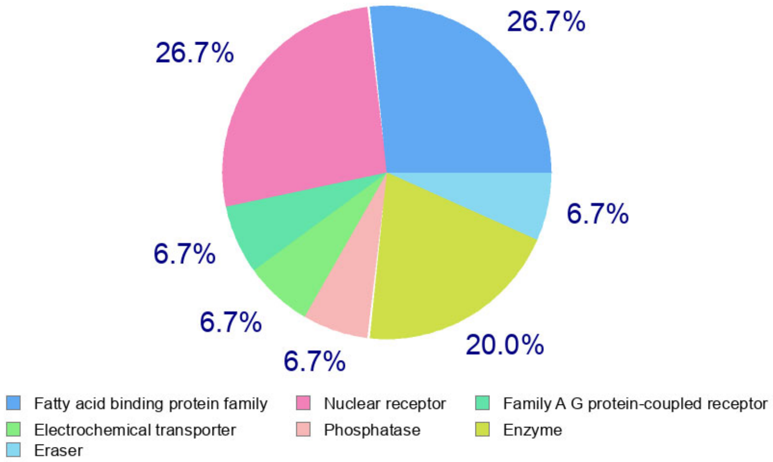

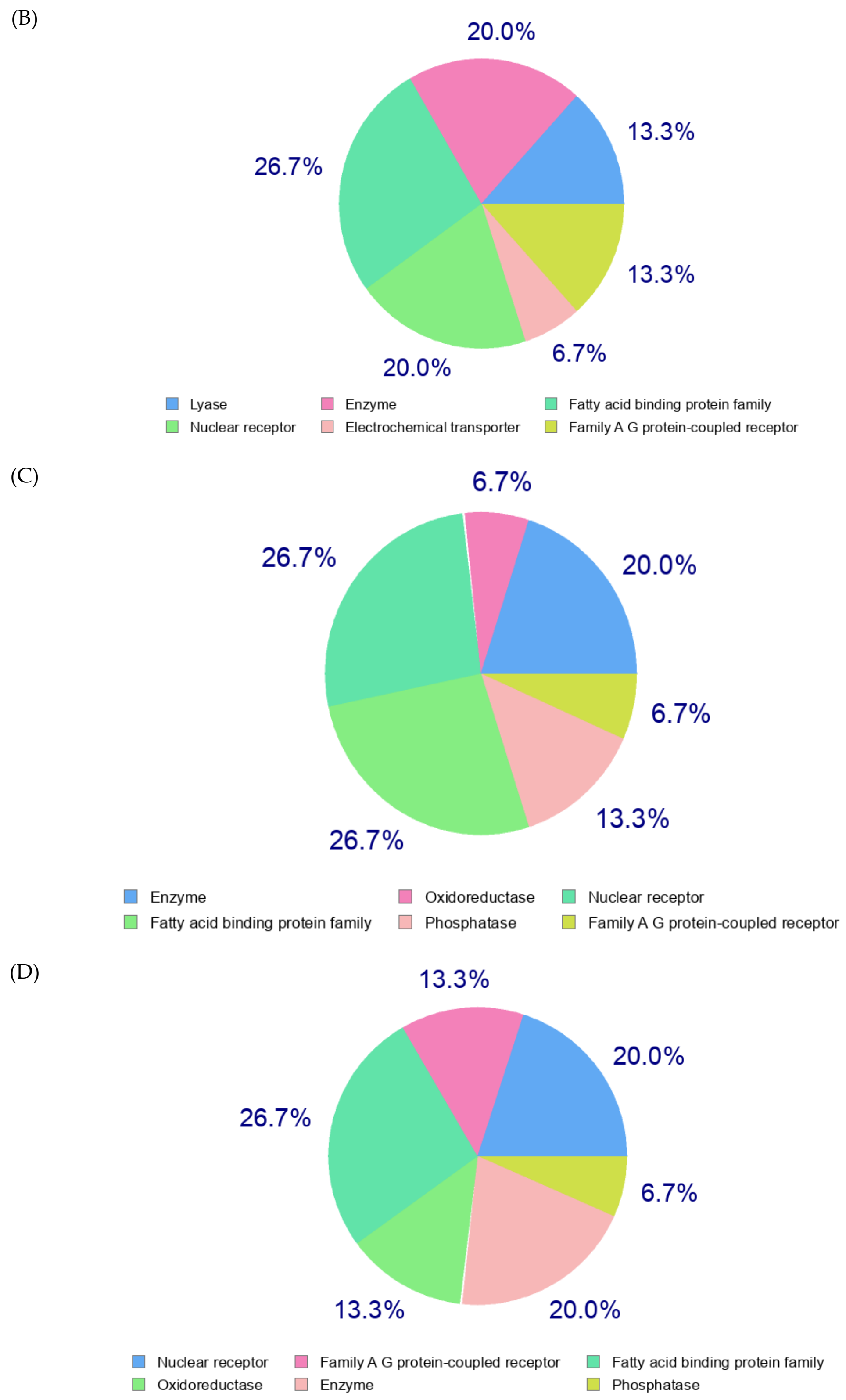
(B)
20.0%
13.3%
26.7%
13.3%
6.7%
20.0%
Lyase
Enzyme
Fatty acid binding protein family
Nuclear receptor
Electrochemical transporter
Family A G protein-coupled receptor
(C)
6.7%
26.7%
20.0%
6.7%
13.3%
26.7%
Enzyme
Oxidoreductase
Nuclear receptor
Fatty acid binding protein family
Phosphatase
Family A G protein-coupled receptor
(D)
13.3%
20.0%
26.7%
6.7%
13.3%
20.0%
Nuclear receptor
Family A G protein-coupled receptor
Fatty acid binding protein family
Oxidoreductase
Enzyme
Phosphatase

(E)

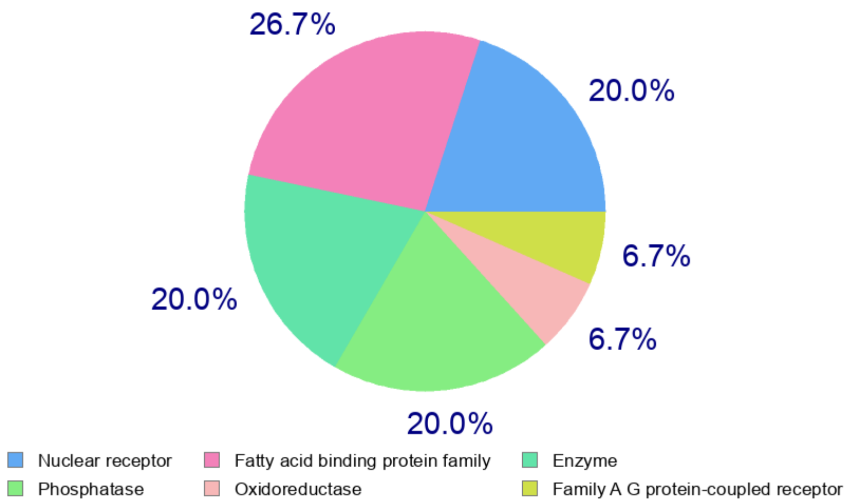

(F)

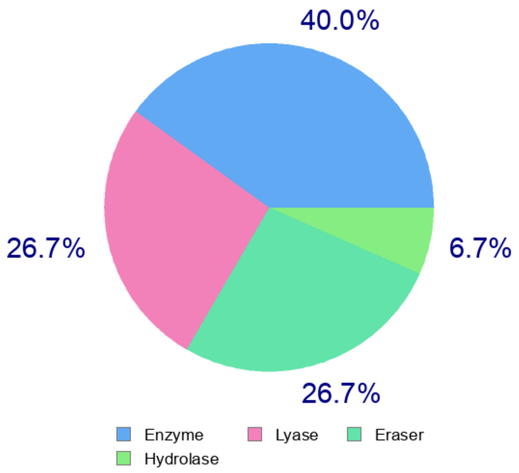

Figure S2. Data on the predicted target of the selected compounds identified in the Ammodaucus leucotrichus methanol extract. The selected compounds, namely (A) n-hexadecanoic acid, (B) hexadecanoic acid methyl ester, (C) 9-octadecenoic acid methyl ester (E)-, (D) (9Z, 12Z)-Octadeca-9,12-dienoic acid, (E) 9-octadecenoic acid, and (F) 2-hydroxyacetohydrazide, exhibit interactions with probable targets, including enzymes, the fatty acid binding protein family, G-protein-coupled receptors, and nuclear receptors.

Table S1. Comprehensive list of the phytochemicals identified in the methanol extract by gas chromatography-mass spectrometry. The table includes compound name, molecular formula, molecular weight, retention time, peak area, and PubChem Compound Identifier (CID).

N°	*Compound*	Classification	*Chemical formula*	*Molecular weight (g/mol)*	*Retention time (min)*	*Peak area (%)*	PubChem CID
S1	9-Octadecenoic acid	Fatty Acid	C18H34O2	282.5	45.880	29.69	637517
S2	Methyl(9Z,12Z)-octadeca-9,12-dienoate	Fatty Acid Ester	C19H36O2	296.5	44.296	8.87	5280590
S3	Octadeca-9,12-dienoic acid	Fatty Acid	C18H32O2	280.4	45.537	8.77	5280450
S4	Methyl hexadecanoate	Fatty Acid Ester	C17H34O2	270.5	38.275	4.87	8181

S5	n-Hexadecanoic acid	Fatty Acid	C16H32O2	256.42	39.713	3.86	985
S6	Methyl (9Z,12Z)-octadeca-9,12-dienoate	Fatty Acid Ester	C19H34O2	294.5	43.994	2.89	5284421
S7	Trichothec-9-ene-3,4,8,15-tetrol, 12,13-epoxy-, 15-acetate 8-(3-methylbutanoate), (3.alpha.,4.beta.,8.alpha.)-	Trichothecene	C22H32O8	424.5	48.948	2.89	520286
S8	Octadecanoic acid	Fatty Acid	C18H36O2	284.5	46.557	2.76	5281
S9	(6Z,9Z,12Z,15Z)-neophytadiene	Sesquiterpene	C20H38	278.5	35.173	1.82	10446
S10	Methyl-5,9,13-trimethyltetradecanoate	Fatty Acid Ester	C18H36O2	284.5	45.295	1.75	554056
S11	Cis-7,8-Epoxy-2-methyloctadecane	Pheromone	C19H38O	282.5	40.551	1.61	205983
S12	3-Isopropyl-6,7-dimethyltricyclo[4.4.0.0(2,8)]decane -9,10-diol	Tricyclic Alcohol	C15H26	238.366	38.410	1.57	491383
S13	2-hydroxyacetohydrazide	Hydrazide	C2H6N2O 2	90.08	2.240	1.53	350536
S14	Phthalic acid, butyl hept-4-yl ester	Phthalate Ester	C19H28O4	320.4	38.269	1.45	91720764
S15	Kauren-19-yl-acetate	Diterpene Ester	C22H34O2	330	44.870	1.31	537596
S16	4,4-Dimethyl-2-pentanone	Ketone	C7H14O	114.19	2.134	1.36	11546
S17	trans-(R,R)-chrysanthemyl (R)-2-methylbutanoate	Ester	C15H26O2	238.37	48.635	1.17	91693481
S18	3-Isopropyl-6,7-dimethyltricyclo[4.4.0.0(2,8)]decane -9,10-diol	Tricyclic Alcohol	C15H26O2	238.37	32.642	0.97	565273
S19	Phthalic acid, tetradecyl trans-dec-3-enyl ester	Phthalate Ester	C32H52O4	500.8	36.002	0.97	91719824
S20	5-Hydroxymethylfurfural	Furfural Derivative	C6H6O3	126.11	18.004	0.87	237332
S21	(1S,2R,4aS,8aS)-1,2,4a,5,8,8a-hexahydro-1,4a-dimethyl-7-(1-methylethyl)-1-naphthalenol	Sesquiterpene Alcohol	C15H24O	220.35	35.490	0.76	10230303 0
S22	8-Isopropyl-1,5-dimethyltricyclo[4.4.0.02,7]dec-4-en-3-one	Tricyclic Ketone	C15H22O	218.33	35.345	0.75	12313013
S23	3,7,11,15-Tetramethyl-2-hexadecen-1-ol	Sesquiterpene Alcohol	C20H40O	296.5	36.591	0.68	5366244
S24	1-Tetracontane	Saturated Hydrocarbon	C40H82	563.1	47.624	0.68	20149

S25	5-nitrobenzofuran-2(3H)-one	Nitrobenzofuran	C8H3NO5	193.11	36.288	0.64	230976
S26	6-(3-Hydroxyprop-1-en-2-yl)-4,8α-dimethyl-2,4α,5,6,7,8-hexahydro-1H-naphthalen-2-ol	Naphthalene Derivative	C15H24O2	236.35	43.610	0.64	535256
S27	(1R,3R,4S,7S)-3,7-dimethyl-1-(propan-2-yl)bicyclo[4.4.0]dec-3-ene-2,6-diol	Sesquiterpene Alcohol	C15H24O	220.35	32.184	0.63	102303030
S28	(2R,5S)-5-[(1R)-1,5-dimethylhex-4-en-1-yl]-2-methylcyclohex-2-en-1-one	Sesquiterpene Aldehyde	C15H24O	220.35	34.931	0.60	565584
S29	1H-Cycloprop[e]azulen-7-ol, decahydro-1,1,7-trimethyl-4-methylene-, [1ar-(1a.alpha.,4a.alpha.,7.beta.,7a.beta.,7b.alpha.)]-	Sesquiterpene Alcohol	C15H24O	220.35	34.590	0.59	6432640
S30	2,4,7,14-Tetramethyl-4-vinyl-tricyclo[5.4.3.0(1,8)]tetradecan-6-ol	Sesquiterpene Alcohol	C20H34O	290.5	35.872	0.59	590916
S31	2-(trans-2,6,6-Trimethylbicyclo[3.3.1]heptan-3-yl)buta-1,3-diene	Terpene	C14H22	190	45.116	0.58	13895
S32	2-aminopropan-1-ol	Amino Alcohol	C3H9NO	75.11	2.205	0.57	5126
S33	(1R,4R,6R,10S)-4,12,12-Trimethyl-9-methylene-5 oxatricyclo[8.2.0.04,6]dodecane	Tricyclic Terpene	C15H24O	220.35	30.995	0.55	1742210
S34	Octahydro-2-benzothiophene	Polycyclic Aromatic Hydrocarbon (PAH)	C20H20O	276.4	35.940	0.54	155017
S35	n-tetradecanoic acid	Fatty Acid	C14H28O2	228.37	34.215	0.49	11005
S36	Methyl 16-R/S-hydroxy-cleroda-3,13(14)-Z-dien-15,16-olide	Diterpene	C22H32O4	360.5	35.014	0.47	16042541
S37	3beta-Trimethylsiloxy-5alpha,6alpha-epoxycholestane	Steroid	C30H54O2Si	474.8	32.394	0.43	16082384
S38	Methyl hexadecanoate	Fatty Acid Ester	C17H34O2	270.5	38.736	0.41	8181
S39	1-dodecyltrichlorosilane	Organosilicon Compound	C12H25Cl3Si	303.8	32.610	0.38	20568
S40	4-Isopropyl-1,7,11-trimethyl-2,7,11-cyclotetradecatrien-1-ol	Not available	C20H34O	290.5	32.330	0.39	5363523

S41	3-Hydroxy-3-methyl-butyric acid, hydrazide	Hydrazide	C5H12N2O2	132.16	35.075	0.38	299142
S42	4-Prop-1-en-2-ylcyclohexene-1-carboxylic acid	Carboxylic Acid	C10H14O	150.22	19.592	0.35	16441
S43	2-Ethylidene-1,7,7-trimethylbicyclo[2.2.1]heptane	Terpene	C12H20	164.29	34.099	0.33	143908
S44	octadecamethyl-1,2,3,4,5,6,7,8,9-nonasiloxane	Siloxane	C18H54O9Si9	667.4	36.130	0.33	11172
S45	Spiro[5.6]dodecan-7-one	Ketone	C12H20O	180.29	32.535	0.30	282264
S46	1-(1,5-Dimethylhexyl)-10-hydroxy-3a,6,6,9a,11a-pentamethylhexadecahydrocyclopenta[7,8]- phenanthro[8a,9-b]oxiren-7-yl acetate	Terpene Ester	C32H54O4	502.8	34.350	0.31	541562
S47	(2R,5S)-5-[(1R)-1,5-dimethylhex-4-en-1-yl]-2-methylcyclohex-2-en-1-one	Sesquiterpene Aldehyde	C15H24	220.350	37.249	0.30	103883460
S48	Heptadecanoic acid	Fatty Acid	C17H34O2	270.5	43.056	0.29	10465
S49	4-Prop-1-en-2-ylcyclohexene-1-carboxylic acid	Carboxylic Acid	C10H14O2	166.22	24.881	0.28	1256
S50	1,2,3,4,5-Penta-O-acetyl-D-xylitol	Xylitol Derivative	C15H22O10	362.33	36.225	0.27	219891
S51	2-isopropyl-5-methylphenol	Phenol Derivative	C10H14O	150.22	20.286	0.25	6989
S52	4'-Hydroxybutyrophenone oxime	Phenolic Oxime	C10H13NO2	179.22	32.775	0.22	135822598
S53	3,5-Dihydroxy-6-methyl-2,3-dihydropyran-4-one	Phenol Derivative	C6H8O4	144.12	14.971	0.20	119838
S54	Formic acid, undecyl ester	Formate Ester	C12H24O2	200.32	26.082	0.20	229382
S55	Cyclopentanol	Cycloalkanol	C5H10O	86.13	24.533	0.19	7298
S56	2,2-bis(oxidanylidene)-1,5-dihydroimidazo[4,5-c][1,2,6]thiadiazin-4-one	Imidazothiadiazinone	C4H4N4O3S	188.17	32.245	0.19	12413165
S57	Spiro[5.5]undeca-1,7-diene	Diene	C11H16	148.24	33.340	0.15	590470
S58	[(Z)-4-Methylsulfonyloxybut-2-enyl] 2-(tert-butoxycarbonylamino)acetate	Ester	C12H21NO7S	323.36	24.495	0.13	5365168
S59	2H-Quinolizine-1-methanol, octahydro-	Quinolizine Derivative	C10H19NO	169.26	34.510	0.13	297272
S60	Fumaric acid, 3,5-dimethylphenyl cyclohexylmethyl ester	Fumarate Ester	C19H24O4	316.4	34.485	0.09	91711292

Table S2. Comprehensive list of the phytochemicals identified in the n-hexane extract by gas chromatography-mass spectrometry. The table includes compound name, molecular formula, molecular weight, retention time, peak area, and PubChem Compound Identifier (CID).

N°	*Compound*	Classification	*Chemical formula*	*Molecular weight (g/mol)*	*Retention time (min)*	*Peak area (%)*	PubChem CID
S1	*9-Octadecenoic acid*	*Fatty Acid*	*C18H34O2*	*282.5*	*45.998*	*29.17*	637517
S2	*Octadeca-9,12-dienoic acid*	*Fatty Acid*	*C18H32O2*	*280.4*	*45.652*	*12.49*	5280450
S3	*n-Hexadecanoic acid*	*Fatty Acid*	*C16H32O2*	*256.42*	*39.938*	*11.81*	985
S4	*Methyl(9Z,12Z)-octadeca-9,12-dienoate*	*Ester*	*C19H36O2*	*296.5*	*44.412*	*5.36*	5280590
S5	*Octadecanoic acid*	*Fatty Acid*	*C18H36O2*	*284.5*	*46.660*	*4.17*	5281
S6	*3-Isopropyl-6,7-dimethyltricyclo[4.4.0.(2,8)]decane-9,10-diol*	*Terpenoid*	*C15H26O2*	*238.37*	*32.416*	*3.96*	565273
S7	*Cyclohexanol, 3-ethenyl-3-methyl-2-(1-methylethenyl)-6-(1-methylethyl)-, (1R,2S,3S,6S)-*	*Alcohol*	*C15H26O*	*222.37*	*32.190*	*3.63*	101316861
S8	*4-Prop-1-en-2-ylcyclohexene-1-carbaldehyde*	*Aldehyde*	*C10H14O*	*150.22*	*15.435*	*3.02*	16441
S9	*Citronellyl tiglate*	*Ester*	*C15H26O2*	*238.37*	*35.032*	*2.53*	6386037
S10	*Isospathulenol*	*Alcohol*	*C15H24O*	*220.35*	*35.430*	*2.34*	14038848
S11	*Spathulenol*	*Alcohol*	*C15H24O*	*220.35*	*34.504*	*2.02*	92231
S12	*Methyl (9Z,12Z)-octadeca-9,12-dienoate*	*Ester*	*C19H34O2*	*294.5*	*44.135*	*1.63*	5284421
S13	*Methyl hexadecanoate*	*Ester*	*C17H34O2*	*270.5*	*38.723*	*1.27*	8181
S14	*3-Methylheptane*	*Hydrocarbon*	*C8H18*	*114.23*	*2.024*	*1.22*	11519
S15	*5,5-diethyl-4-methyl-6-spiro[2.3]hexan*	*Hydrocarbon*	*C11H18O*	*166.26*	*22.783*	*1.05*	550247
S16	*6-Methyl-1,2,3,5,8,8a-hexahydronaphthalene*	*Hydrocarbon*	*C11H16*	*148.24*	*33.025*	*0.88*	562093
S17	*2,6-Dimethoxy-4-(prop-2-en-1-yl)phenol*	*Phenol*	*C11H14O3*	*194.23*	*29.040*	*0.79*	226486
S18	*(3,7,7-Trimethyl-bicyclo[2.2.1]hept-2-yl)-methanol*	*Alcohol*	*C11H20O*	*168.28*	*34.191*	*0.75*	558050
S19	*Stigmastan-6,22-dien, 3,5-dedihydro-*	*Sterol*	*C29H46*	*394.7*	*45.088*	*0.74*	5364573
S20	*1,4-Dimethyl-7-(prop-1-en-2-yl)-1,2,3,4,5,6,7,8-octahydroazulene*	*Terpenoid*	*C15H24*	*204.35*	*31.183*	*0.71*	5317844
S21	*2(3H)-Benzofuranone, 6-ethenylhexahydro-3,6-dimethyl-5-(1-methylethenyl)-*	*Lactone*	*C15H22O2*	*234.33*	*34.577*	*0.65*	14038380

S22	*3-Isopropyl-6,7-dimethyltricyclo[4.4.0.(2,8)]decane-9,10-diol*	*Terpenoid*	*C15H26O2*	*238.37*	*39.430*	*0.62*	565273
S23	*Cholest-5-en-3-ol (3.beta.)-, propanoate*	*Steroid*	*C30H50O2*	*442.7*	*43.595*	*0.59*	12451
S24	*2(3H)-Benzofuranone, 6-ethenylhexahydro-3,6-dimethyl-5-(1-methylethenyl)-*	*Lactone*	*C15H22O2*	*234.33*	*35.483*	*0.58*	14038380
S25	*Myristic acid*	*Fatty Acid*	*C14H28O2*	*228.37*	*33.676*	*0.44*	11005
S26	*2-Isopropyl-4a-methyl-8-methylenedecalin-1,5-diol*	*Terpenoid*	*C15H26O2*	*238.37*	*37.755*	*0.42*	14137570
S27	*Cyclohexanol, 3-ethenyl-3-methyl-2-(1-methylethenyl)*	*Alcohol*	*C15H26O*	*222.37*	*25.630*	*0.36*	101316861
S28	*2,2-Dimethylhexane*	*Hydrocarbon*	*C8H18*	*114.23*	*2.204*	*0.34*	11551
S29	*Gamma-dodecalactone*	*Lactone*	*C12H22O2*	*198.3*	*31.423*	*0.34*	16821
S30	*1H-cycloprop(e)azulen-7-ol, decahydro-1,1,7-trimethyl-4-methylene-(1AR,4AR,7S,7AR,7BR)-*	*Terpenoid*	*C15H24O*	*220.35*	*28.699*	*0.32*	92231
S31	*1,3,5-Trimethyl-2,6-di(cyclopentyl)benzene*	*Hydrocarbon*	*C19H28*	*256.399*	*42.018*	*0.30*	620628
S32	*1,1,4,7tetramethyldecahydro-1H-cyclopropa(e)azulen-4-ol*	*Terpenoid*	*C15H26O*	*222.37*	*32.623*	*0.27*	11996452
S33	*2(3H)-Furanone, 5-hexyldihydro-*	*Lactone*	*C10H18O2*	*170.25*	*23.746*	*0.26*	12813
S34	*2-(Tricyclo[3.3.1.13,7]dec-1-yl)propanoic acid*	*Carboxylic Acid*	*C13H18O2*	*206.28*	*32.311*	*0.26*	609874
S35	*2-Naphthalenemethanol, decahydro-.al*	*Alcohol*	*C15H26O*	*222.37*	*30.962*	*0.25*	91457
S36	*Juniper camphor (1,4a-dimethyl-7-propan-2-ylidene-3,4,5,6,8,8a-hexahydro-2H-naphthalen-1-ol)*	*Terpenoid*	*C15H26O*	*222.37*	*31.297*	*0.24*	521214
S37	*Taylorione (4-[(1S,3R)-2,2-dimethyl-3-(5-methylidenecyclopenten-1-yl)cyclopropyl]butan-2-one)*	*Terpenoid*	*C15H22O*	*218.33*	*40.235*	*0.24*	12152394
S38	*Sinulariolide ((8Z)-12-hydroxy-5,9,13-trimethyl-16-methylidene-4,14-dioxatricyclo[11.3.2.03,5]octadec-8-en-15-one)*	*Macrolide*	*C20H30O4*	*334.4*	*39.252*	*0.22*	5358848
S39	*1-((1S,3aR,4R,7S,7aS)-4-Hydroxy-7-isopropyl-4-methyloctahydro-1H-inden-1-yl)ethan-1-one*	*Ketone*	*C15H26O2*	*238.37*	*33.324*	*0.21*	10466745
S40	*1,1,4,7-Tetramethyldecahydro-1H-cyclopropa[e]azulen-7-ol*	*Terpenoid*	*C15H26O*	*222.37*	*35.633*	*0.21*	11996452

S41	*4-Isobenzofuranol, octahydro-3a,7a-dimethyl-*	*Alcohol*	*C10H18O2*	*170.25*	*36.541*	*0.21*	41057
S42	*(1S,3aS,4S,5S,7aR,8R)-5-Isopropyl-1,7a-dimethyloctahydro-1H-1,4-methanoinden-8-ol*	*Terpenoid*	*C15H26O*	*222.37*	*29.866*	*0.20*	12303891
S43	*2-Oxabicyclo[4.2.0]oct-4-en-3-one, rel*	*Ketone*	*C28H22F2O6*	*492.5*	*48.336*	*0.20*	5377136
S44	*Platambin (4a-methyl-8-methylidene-2-propan-2-yl-1,2,3,4,5,6,7,8a-octahydronaphthalene-1,5-diol)*	*Terpenoid*	*C15H26O2*	*238.37*	*37.086*	*0.19*	613194
S45	*(1R,7S,E)-7-Isopropyl-4,10-dimethylenecyclodec-5-enol*	*Terpenoid*	*C15H24O*	*220.35*	*31.944*	*0.15*	13304974
S46	*(E)-Valerenyl isovalerate*	*Ester*	*C20H32O2*	*304.5*	*40.978*	*0.15*	91730081
S47	*Dodecanoic acid*	*Fatty Acid*	*C12H24O2*	*200.32*	*28.066*	*0.14*	3893
S48	*1-Nonadecene*	*Alkene*	*C19H38*	*266.5*	*40.815*	*0.11*	29075
S49	*(S)-(-)-(4-Isopropenyl-1-cyclohexenyl)methanol*	*Alcohol*	*C10H16O*	*152.23*	*18.261*	*0.11*	369312
S50	*4-(1-Methylvinyl)cyclohex-1-ene-1-ethyl formate*	*Ester*	*C12H18O2*	*194.27*	*21.755*	*0.10*	3023295
S51	*Spathulenol ((1aR,4aR,7S,7aR,7bR)-1,1,7-trimethyl-4-methylidene-1a,2,3,4a,5,6,7a,7b-octahydrocyclopropa[h]azulen-7-ol)*	*Alcohol*	*C15H24O*	*220.35*	*33.549*	*0.10*	92231
S52	*1-Naphthalenepropanol, .alpha.-ethenyldecahydro-2-hydroxy-alpha, 2,5,5,8a-pentamethyl*	*Terpenoid*	*C20H36O2*	*308.5*	*36.901*	*0.10*	163263
S53	*1,2-Cyclohexanediol, 1-methyl-4-(1-methylethenyl)*	*Alcohol*	*C10H18O2*	*170.25*	*20.427*	*0.09*	441246
S54	*3-Methylhexane*	*Hydrocarbon*	*C7H16*	*100.2*	*2.119*	*0.08*	11507
S55	*(3E)-6-Methyl-3,5-heptadien-2-one*	*Ketone*	*C8H12O*	*124.18*	*22.650*	*0.08*	5370101
S56	*Tricyclo[4.4.0.0(2,7)]dec-8-ene-3-methanol*	*Terpenoid*	*C15H24O*	*220.35*	*27.448*	*0.08*	521030
S57	*1-Nonadecene*	*Alkene*	*C19H38*	*266.5*	*34.896*	*0.08*	29075
S58	*(Z)-Dodec-5-en-4-olide*	*Lactone*	*C12H20O2*	*196.29*	*43.401*	*0.08*	67469926
S59	*3-Cyclohexene-1-carboxaldehyde*	*Aldehyde*	*C7H10O*	*110.15*	*2.459*	*0.05*	7508
S60	*4-Isopropenylcyclohexanone*	*Ketone*	*C9H14O*	*138.21*	*10.440*	*0.05*	549291
S61	*Heptane*	*Hydrocarbon*	*C7H16*	*100.2*	*2.329*	*0.04*	8900

Table S3. Comparison of the key phytocomponents (with their peak area) present in the methanol and n-hexane extracts, identified by gas chromatography/mass spectrometry.

N°	Methanol extraction		n-Hexane extraction	
	Compounds (IUPAC Name)	*Peak area (%)*	Compounds (IUPAC Name)	*Peak area (%)*
1	9-Octadecenoic acid	29.69	9-Octadecenoic acid	29.17
2	Methyl(9Z,12Z)-octadeca-9,12-dienoate	8.87	Octadeca-9,12-dienoic acid	12.49
3	Octadeca-9,12-dienoic acid	8.77	n-Hexadecanoic acid	11.81
4	Methyl hexadecanoate	4.87	Methyl(9Z,12Z)-octadeca-9,12-dienoate	5.36
5	n-Hexadecanoic acid	3.86	Octadecanoic acid	4.17
6	Methyl (9Z,12Z)-octadeca-9,12-dienoate	2.89	3-Isopropyl-6,7-dimethyltricyclo [4.4.0.(2,8)]decane-9,10-diol	3.96
7	Trichothec-9-ene-3,4,8,15-tetrol, 12,13-epoxy-, 15-acetate 8-(3-methylbutanoate), (3.alpha.,4.beta.,8.alpha.)-	2.89	Cyclohexanol, 3-ethenyl-3-methyl-2-(1-methylethenyl)-6-(1-methylethyl)-, (1R,2S,3S,6S)-	3.63
8	Octadecanoic acid	2.76	4-Prop-1-en-2-ylcyclohexene-1-carbaldehyde	3.02
9	Neophytadiene	1.82	Citronellyl tiglate	2.53
10	Methyl-5,9,13-trimethyltetradecanoate	1.75	Isospathulenol	2.34
11	Disparlure	1.61	Spathulenol	2.02
12	3-Isopropyl-6,7-dimethyltricyclo [4.4.0.0(2,8)]decane-9,10-diol	1.57	Methyl (9Z,12Z)-octadeca-9,12-dienoate	1.63
13	2-hydroxyacetohydrazide	1.53	Methyl hexadecanoate	1.27
14	Phthalic acid, butyl hept-4-yl ester	1.45	3-Methylheptane	1.22
15	2-Pentanone, 4,4-dimethyl-	1.36	5,5-diethyl-4-methyl-6-spiro[2.3]hexan	1.05
	Sum	76.10	Sum	85.67

Table S4: Impact of different concentrations of the methanol and n-hexane extracts from *Ammodaucus leucotrichus* seeds on BSA denaturation. Comparison with diclofenac (reference anti-inflammatory drug) in a BSA denaturation assay and IC_{50} values (µg/ml) for BSA denaturation inhibition. Data are the mean ± standard error of the mean.

Active ingredients	Concentration (µg/mL)	Log concentration (µg/mL)	Inhibition (%)	IC_{50} (µg/mL)
Methanol extract	3.00	0.59	5.67 ±0.50	5408.00
	7.81	0.89	13.67 ±0.50	
	15.62	1.19	14.67 ±0.50	
	31.20	1.49	17.67 ±0.50	
	62.50	1.79	22.00 ±0.00	
	125.00	2.09	24.67 ±0.50	
	250.00	2.39	26.00 ±0.00	
n-hexane extract	3.00	0.59	8.30 ±0.45	14.30
	7.81	0.89	20.93 ±0.32	

	15.62	1.19	56.14 ±0.05	
	31.20	1.49	84.05 ±0.45	
	62.50	1.79	90.36 ±0.57	
	125.00	2.09	91.33 ±0.57	
	250.00	2.39	94.00 ±0.00	
Diclofenac	3.00	0.59	10.67 ±0.15	42.30
	7.81	0.89	24.67 ±0.50	
	15.62	1.19	30.00 ±0.00	
	31.20	1.49	34.10 ±0.49	
	62.50	1.79	51.36 ±0.90	
	125.00	2.09	76.85 ±0.57	
	250.00	2.39	99.22 ±0.41	

Table S5: Trypsin inhibition by the methanol and n-hexane extracts from *Ammodaucus leucotrichus* seeds compared with Diclofenac (reference drug) at different concentrations, and IC_{50} values (µg/ml) for trypsin inhibition. Data are the mean ± standard error of the mean.

Active ingredients	Concentration (µg/mL)	Log concentration (µg/mL)	Inhibition (%)	IC_{50} (µg/mL)
Methanol extract	31.20	1.49	3.00 ±0.00	82.97
	62.50	1.79	23.00±0.00	
	125.00	2.09	85.00±0.57	
	250.00	2.39	100.00±0.01	
	500.00	2.69	100.00 ±0.01	
n-Hexane extract	31.20	1.49	6.00±0.00	202.70
	62.50	1.79	10.33±0.57	
	125.00	2.09	30.00±0.00	
	250.00	2.39	51.00±0.48	
	500.00	2.69	100.00±0.00	
Diclofenac	31.20	1.49	6.00±0.00	97.04
	62.50	1.79	23.00±0.00	
	125.00	2.09	64.67±0.57	
	250.00	2.39	100.00±0.00	
	500.00	2.69	100.00±0.00	

Table S6: Binding energies (in kJ/mol) of the 59 key compounds identified in the methanol extract from *Ammodaucus leucotrichus* seeds and of diclofenac during their interactions with trypsin. The different binding energy values reflect variations in the inhibitory potential of the compounds against trypsin.

N°	Compound	Classification	PubChem CID	Binding energy (KJ/mol)
S1	2-hydroxyacetohydrazide	Hydrazide	350536	-17.13

S2	3-Hydroxy-3-methyl-butyric acid, hydrazide	Hydrazide	299142	-14.44
S3	2,2-bis(oxidanylidene)-1,5-dihydroimidazo[4,5-c][1,2,6]thiadiazin-4-one	Imidazo Thiadia-zinone	12413165	-14.39
S4	4'-Hydroxybutyrophenone oxime	Oxime	135822598	-13.45
S5	Diclofenac (2-[(2,6-dichlorophenyl)amino]benzeneacetic acid)		3033	-13.01
S6	3,5-Dihydroxy-6-methyl-2,3-dihydropyran-4-one	Pyranone	119838	-12.41
S7	6-(3-Hydroxyprop-1-en-2-yl)-4,8α-dimethyl-2,4α,5,6,7,8-hexahydro-1H-naphthalen-2-ol	Alcohol	535256	-11.44
S8	5-Hydroxymethylfurfural	Furfural	237332	-11.21
S9	5-nitrobenzofuran-2(3H)-one	Benzofuranone	230976	-9.89
S10	1-Propanol, 2-amino-, (.+/-.)-	Amino Alcohol	5126	-8.28
S11	(7S)-1,1,7-Trimethyl-4-methylidene-1α,2,3,4α,5,6,7α,7β-octahydrocyclopropa[h]azulen-7-ol	Sesquiterpene Alcohol	6432640	-8.01
S12	Spiro[5.6]dodecan-7-one	Ketone	282264	-7.27
S13	(1R,4R,6R,10S)-4,12,12-Trimethyl-9-methylene-5-oxatricyclo[8.2.0.04,6]dodecane	Triterpene	1742210	-5.99
S14	Cyclopentanol	Alcohol	7298	-5.83
S15	Thymol (2-isopropyl-5-methylphenol.)	Phenol	6989	-5.51
S16	Isospathulenol ((1R,4R,6R)-1,4,7,7-tetramethylbicyclo[4.1.0]heptan-2-ol)	Sesquiterpene Alcohol	102303030	-5.17
S17	Fumaric acid (3,5-dimethylphenyl cyclohexylmethyl ester)	Fumarate Ester	91711292	-4.87
S18	4-Prop-1-en-2-ylcyclohexene-1-carboxylic acid	Carboxylic Acid	1256	-4.82
S19	2H-Quinolizine-1-methanol, octahydro-	Quinolizine Al-cohol	297272	-4.67
S20	8-Isopropyl-1,5-dimethyltricyclo[4.4.0.02,7]dec-4-en-3-one	Ketone	12313013	-4.74
S21	Formic acid undecyl ester (Undecyl methanoate)	Carboxylic Acid Ester	229382	-4.19
S22	[(Z)-4-Methylsulfonyloxybut-2-enyl] 2-(tert-butoxycarbonylamino)acetate	Ester	5365168	-4.17
S23	Phthalic acid, butyl hept-4-yl ester	Phthalate Ester	91720764	-3.19
S24	trans-(R,R)-chrysanthemyl (R)-2-methylbutanoate	Terpenoid Ester	91693481	-2.10
S25	4,4-dimethylpentan-2-one	Ketone	11546	-2.00
S26	2-Ethylidene-1,7,7-trimethylbicyclo[2.2.1]heptane	Bicyclic Hydro-carbon	143908	-1.51
S27	Thunbergol (4-Isopropyl-1,7,11-trimethyl-2,7,11-cyclotetradecatrien-1-ol)	Terpenoid	5363523	-1.39

S28	Methyl 16-R/S-hydroxy-cleroda-3,13(14)-Z-dien-15,16-olide	Diterpenoid Lactone	16042541	-0.56
S29	3beta-Trimethylsiloxy-5alpha,6alpha-epoxycholestane	Steroid	16082384	0
S30	Trichloro(dodecyl)silane	Organosilane	20568	0
S31	3-Isopropyl-6,7-dimethyltricyclo[4.4.0.0(2,8)]decane-9,10-diol	Sesquiterpene Alcohol	565273	0
S32	Spiro[5.5]undeca-1,7-diene (1,1,7,7-tetracyclo[6.4.0.0^{2},^{6}.0^{7},^{11}]dodeca-2,4,6,8,10-pentaene)	Hydrocarbon	590470	0
S33	n-tetradecanoic acid	Fatty Acid	11005	0
S34	1-(1,5-Dimethylhexyl)-10-hydroxy-3a,6,6,9a,11a-pentamethylhexadecahydrocyclopenta[7,8]-phenanthro[8a,9-b]oxiren-7-yl acetate	Sesquiterpene Ester	541562	0
S35	Longifolenaldehyde (2R,5S)-5-[(1R)-1,5-dimethylhex-4-en-1-yl]-2-methylcyclohex-2-en-1-one)	Aldehyde	565584	0
S36	Neophytadiene ((6Z,9Z,12Z,15Z)-neophytadiene)	Sesquiterpene	10446	0
S37	2,4,7,14-Tetramethyl-4-vinyl-tricyclo[5.4.3.0(1,8)]tetradecan-6-ol	Terpenoid Alcohol	590916	0
S38	Benzo - octahydro -acephenanthrylene	Hydrocarbon	155017	0
S39	Phthalic acid, tetradecyl trans-dec-3-enyl ester	Phthalate Ester	91719824	0
S40	Octadecamethyl-cyclononasiloxane	Siloxane	11172	0
S41	1,2,3,4,5-Penta-O-acetyl-D-xylitol	Xylitol Ester	219891	0
S42	4-Prop-1-en-2-ylcyclohexene-1-carbaldehyde	Aldehyde	16441	0
S43	3,7,11,15-Tetramethyl-2-hexadecen-1-ol	Terpenoid Alcohol	5366244	0
S44	Longifolenaldehyde ((2R,5S)-5-[(1R)-1,5-dimethylhex-4-en-1-yl]-2-methylcyclohex-2-en-1-one)	Aldehyde	103883460	0
S45	Cyclopropanebutanoic acid, Methyl-4-[2-[[2-[[2-[(2-pentylcyclopropyl)methyl]cyclopropyl]methyl]cyclopropyl]methyl]-cyclopropyl]butanoate	Cyclopropane Carboxylic Acid Ester	554084	0
S46	Methyl hexadecanoate	Fatty Acid Ester	8181	0
S47	n-Hexadecanoic acid	Fatty Acid	985	0
S48	Disparlure (cis-7,8-epoxy-2-methyloctadecane)	Pheromone	205983	0
S49	Heptadecanoic acid	Fatty Acid	10465	0
S50	Methyl (9Z,12Z)-octadeca-9,12-dienoate	Fatty Acid Ester	5284421	0
S51	Methyl(9Z,12Z)-octadeca-9,12-dienoate	Fatty Acid Ester	5280590	0
S52	Kauren-19-yl-acetate	Diterpene Ester	5284421	0
S53	2-(trans-2,6,6-Trimethylbicyclo[3.3.1]heptan-3-yl)buta-1,3-diene	Terpenoid Diene	5280590	0
S54	Methyl-5,9,13-trimethyltetradecanoate	Fatty Acid Ester	554056	0
S55	Octadeca-9,12-dienoic acid	Fatty Acid	5280450	0

S56	9-Octadecenoic acid	Fatty Acid	637517	0
S57	Octadecanoic acid	Fatty Acid	5281	0
S58	Tetracontane	Hydrocarbon	20149	0
S59	Trichothec-9-ene-3,4,8,15-tetrol, 12,13-epoxy-, 15-acetate 8-(3-methylbutanoate), (3.alpha.,4.beta.,8.alpha.)-	Trichothecene Mycotoxin	520286	0

Table S7. PASS prediction of the bioactivities of the 59 phytochemicals identified in the methanol extract from *Ammodaucus leucotrichus* seeds.

N°	Compound	Anti-inflammatory effects		RA therapeutic target
		Pa	Pi	
S1	Tetradecanoic acid	0.935	0.003	Sphinganine kinase inhibitor
S2	Octadecanoic acid	0.935	0.003	
S3	Heptadecanoic acid	0.935	0.003	
S4	n-Hexadecanoic acid	0.935	0.003	
S5	Tetracontane	0.915	0.004	
S6	Methyl(9Z,12Z)-octadeca-9,12-dienoate	0.905	0.004	
S7	9-Octadecenoic acid	0.905	0.002	
S8	Octadeca-9,12-dienoic acid	0.873	0.005	
S9	2-Pentanone, 4,4-dimethyl-	0.867	0.004	
S10	Phthalic acid, tetradecyl trans-dec-3-enyl ester	0.866	0.007	
S11	Methyl hexadecanoate	0.845	0.009	
S12	Formic acid, undecyl ester	0.819	0.012	
S13	Trichloro(dodecyl)silane	0.814	0.012	
S14	1,2,3,4,5-Penta-O-acetyl-D-xylitol	0.819	0.012	
S15	Disparlure	0.818	0.012	
S16	Phthalic acid, butyl hept-4-yl ester	0.754	0.018	
S17	Methyl (9Z,12Z)-octadeca-9,12-dienoate	0.648	0.029	
S18	Cyclopentanol	0.860	0.004	JAK2 expression inhibitor
S19	Thymol	0.737	0.014	
S20	3,5-Dihydroxy-6-methyl-2,3-dihydropyran-4-one	0.730	0.014	
S21	2-Ethylidene-1,7,7-trimethylbicyclo[2.2.1]heptane	0.724	0.015	
S22	Octahydro-2-benzothiophene	0.715	0.016	
S23	Spiro[5.5]undeca-1,7-diene	0.707	0.017	
S24	Spiro[5.6]dodecan-7-one	0.684	0.019	
S25	3-Isopropyl-6,7-dimethyltricyclo[4.4.0.0(2,8)]decane-9,10-diol	0.571	0.037	

S26	2-(trans-2,6,6-Trimethylbicyclo[3.3.1]heptan-3-yl)buta-1,3-diene	0.868	0.002	Transcription factor NF kappa B stimulant
S27	8-Isopropyl-1,5-dimethyltricyclo[4.4.0.02,7]dec-4-en-3-one	0.735	0.004	
S28	4-Prop-1-en-2-ylcyclohexene-1-carboxylic acid	0.733	0.004	
S29	thunbergol ((2E,7E,11E)-1,7,11-trimethyl-4-propan-2-ylcyclotetradeca-2,7,11-trien-1-ol)	0.730	0.004	
S30	Neophytadiene (7,11,15-trimethyl-3-methylidenehexadec-1-ene)	0.620	0.008	
S31	2,4,7,14-Tetramethyl-4-vinyl-tricyclo[5.4.3.0(1,8)]tetradecan-6-ol	0.563	0.013	
S32	Cyclopropanebutanoic acid, Methyl-4-[2-[[2-[[2-[(2-pentylcyclopropyl)methyl]cyclopropyl]methyl]cyclopropyl]methyl]-cyclopropyl]butanoate	0.388	0.057	
S33	1-amino-propan-2-ol racemate.	0.926	0.003	G-protein-coupled receptor kinase inhibitor
S34	5-Hydroxymethylfurfural	0.749	0.018	
S35	3,7,11,15-Tetramethyl-2-hexadecen-1-ol	0.694	0.023	
S36	2H-Quinolizine-1-methanol, octahydro-	0.680	0.025	
S37	3-Hydroxy-3-methyl-butyric acid, hydrazide	0.656	0.027	
S38	Isospathulenol ((1R,4R,6R)-1,4,7,7-tetramethylbicyclo[4.1.0]heptan-2-ol)	0.729	0.005	MMP9 expression inhibitor
S39	2,2-bis(oxidanylidene)-1,5-dihydroimidazo[4,5-c][1,2,6]thiadiazin-4-one	0.675	0.032	Methylenetetrahydrofolate reductase (NADPH) inhibitor
S40	5-nitrobenzofuran-2(3H)-one	0.878	0.004	Glutathione S-transferase
S41	[(Z)-4-Methylsulfonyloxybut-2-enyl] 2-(tert-butoxycarbonylamino)acetate	0.840	0.006	
S42	Methyl-5,9,13-trimethyltetradecanoate	0.641	0.032	
S43	Octadecamethyl-cyclononasiloxane	0.852	0.004	Complement factor D inhibitor
S44	4'-Hydroxybutyrophenone oxime	0.619	0.034	
S45	Trichothec-9-ene-3,4,8,15-tetrol, 12,13-epoxy-, 15-acetate 8-(3-methylbutanoate), (3.alpha.,4.beta.,8.alpha.)-	0.858	0.002	Immunosuppressant
S46	1-(1,5-Dimethylhexyl)-10-hydroxy-3a,6,6,9a,11a-pentamethylhexadecahydrocyclopenta[7,8]- phenanthro[8a,9-b]oxiren-7-yl acetate	0.795	0.005	
S47	6-(3-Hydroxyprop-1-en-2-yl)-4,8α-dimethyl-2,4α,5,6,7,8-hexahydro-1H-naphthalen-2-ol	0.759	0.010	
S48	(1R,4R,6R,10S)-4,12,12-Trimethyl-9-methylene-5-oxatricyclo[8.2.0.04,6]dodecane	0.758	0.010	
S49	3beta-Trimethylsiloxy-5alpha,6alpha-epoxycholestane	0.727	0.014	
S50	4-Prop-1-en-2-ylcyclohexene-1-carbaldehyde	0.722	0.014	
S51	(7S)-1,1,7-Trimethyl-4-methylidene-1α,2,3,4α,5,6,7α,7β-octahydrocyclopropa[h]azulen-7-ol	0.713	0.015	

S52	Longifolenaldehyde ((2R,5S)-5-[(1R)-1,5-dimethylhex-4-en-1-yl]-2-methylcyclohex-2-en-1-one)	0.713	0.015	
S53	Methyl 16-R/S-hydroxy-cleroda-3,13(14)-Z-dien-15,16-olide	0.675	0.020	
S54	Isospathulenol ((1R,4R,6R)-1,4,7,7-tetramethylbicyclo[4.1.0]heptan-2-ol)	0.658	0.022	
S55	Thunbergol (4-Isopropyl-1,7,11-trimethyl-2,7,11-cyclotetradecatrien-1-ol)	0.613	0.027	
S56	Kauren-19-yl-acetate	0.613	0.027	
S57	trans-(R,R)-chrysanthemyl (R)-2-methylbutanoate	0.473	0.048	
S58	Fumaric acid, 3,5-dimethylphenyl cyclohexylmethyl ester	0.402	0.065	
S59	2-hydroxyacetohydrazide			Carbons 1 < 3
